# Effect of sampling on the descriptive distributions of correlated random walks

**DOI:** 10.1007/s00285-026-02479-y

**Published:** 2026-10-29

**Authors:** Joseph D. Bailey, Jessica Claridge

**Affiliations:** School of Mathematics, Statistics and Actuarial Science, University of Essex, Wivenhoe Park, Colchester, Essex CO4 3SQ UK

**Keywords:** Random walk, Circular statistics, Movement ecology, 92-10, 62P12, 60G50

## Abstract

Random walks are commonly used to model movement throughout the sciences, from the motion of particles and molecules to the observed behaviour of animals and crowds. The correlated random walk, which assumes a level of persistence between movement directions, has become ubiquitous in the analysis and modelling of movement in recent times. Whilst many properties of the correlated random walk are known, there are still many which are not fully understood and, therefore, under-utilised in movement data analysis. Here we consider the effect that sub-sampling has on the descriptive distributions of correlated random walks. Our work demonstrates the connection between the distributions of turning angles and step-lengths that characterise a correlated random walk, along with the resulting distributions found after sub-sampling. We provide examples for where this approach could aid in movement analysis as well as determining future ways the work could be extended.

## Introduction

Random walks (RW) appear throughout applied mathematics and the sciences; from the motion of particles in physical sciences (Patlak 1953; Alt 1980) to the modelling of animal movement in life sciences (Berg 1993; Codling et al. 2008; Bailey et al. 2018), and from predicting financial markets in economics (Fama 1995) to the programming of robotics in computer science (Dimidov et al. 2016).

Early work concerning RW models assumed turns were uniformly distributed on the unit circle. These simple random walks (SRW) give movement akin to simple Brownian motion and, though simplistic, they have been successfully incorporated into many natural and physical processes including in the modelling of gas particles (Weiss and Rubin 1983), solid state physics (Weiss 1983), epidemiology (Fofana and Hurford 2017; White et al. 2018) and ecological phenomena such as dispersal (Skellam 1951; Turchin 1998; Okubo et al. 2001), foraging behaviour (Bell 1990) and predator–prey behaviour (Kareiva 1257). Extensions to the SRW model aim to more closely mimic real-world drivers of movement, such as including attraction/repulsion towards a global direction or location, due to some external or internal force, for example a gradient function from a field force or a reaction to olfactory sense when migrating or navigating towards a food source. This bias in the direction of movement leads to the biased random walk (BRW) model, which has been used in the analysis of many processes including chemotaxis (Alt 1980; Codling et al. 2008), diffusion in anomalous media (Bouchaud and Georges 1990), migration (Saul et al. 2012; Papastamatiou et al. 2013), foraging (Farnsworth and Beecham 1999; Barton et al. 2009), home-range behaviour (Börger et al. 2008; Breed et al. 2017) and the movement of microorganisms (Hill and Häder 1997).

An alternative approach is to consider how the direction of movement at each step changes relative to the direction of the previous step; a correlation in these headings results in the correlated random walk (CRW), which has become a popular null model in animal movement (Fagan and Calabrese 2014). In general, movement is therefore described as a highly correlated, giving near straight line movement, or weakly correlated, in which movement appears more random and similar to a SRW. CRWs have a wide range of applications within ecology including the modelling of group behaviour and movement (Haydon et al. 2008; Codling and Bode 2014), modelling invasive spread of plants (Andersen et al. 2005) and are regularly used in the analysis of individual movement (McClintock and Michelot 2018; Grant et al. 2018; Bailey et al. 2021a). Outside of mathematical ecology, CRW have continued to be utilised and developed in fields including physics (Cressoni et al. 2013; Sadjadi et al. 2015; Yuste et al. 2024), soft matter physics (Liu et al. 2021), materials science (Bonilla and Bhatia 2012) and robotics, Dimidov et al. (2016); Pang et al. (2021).

One natural problem within the RW framework is the question of sampling, which entails selecting a subset of the points in the random walk (i.e. every 10th point) and analysing the subsequent path found by connecting these. An open problem concerns how the sampling rate affects the properties of the random walk. In terms of data analysis, sampling from the observed data is used for a variety of reasons, such as reducing noise (Bailey et al. 2021b), reducing volume of data (Gupte et al. 2022), and allowing for meaningful/appropriate scales of movement e.g. recording the movement of large ungulates at multiple fixes per second will not give reliable results compared to taking values every few minutes (Nathan et al. 2022; Schlägel and Lewis 2016; Potts et al. 2018). However, the effects on the underlying model that this rescaling has is not fully understood, despite the need to ensure the appropriateness of any analyses on such datasets, regardless of scale, being well known (Schlägel and Lewis 2016). As RW models assume that reorientation events occur precisely at the frequency of the recorded data (Codling and Hill 2005), much previous work has concerned the identification of meaningful turning points (reorientation events) across the movement trajectory and the effect that sampling has on the potential to miss or mis-locate such points (Codling and Hill 2005; Nathan et al. 2022).

Concentrating on pure CRW processes, Codling and Hill (2005) considered the effect of summary statistics when a CRW is sampled, demonstrating via large-scale simulation that angular deviation observes a linear relation with the square root of the sampling rate, as well as generating expressions for the mean and variance of the distribution of speeds (analogous to step-lengths); although, they note that this relationship breaks down as the RW becomes closer to a SRW. Rosser et al. (2013) extended this work by further simulation to demonstrate the complex relationship between the sampling rate and the turning angle distributions. They also determine an expression for the joint distribution between step lengths and turning angles when the number of true reorientation events within the sub-sampled RW is unknown, achieving an analytical expression that holds for large sampling rates and for certain constraints on the initial turning angle distribution. Nams (2013), demonstrated through extensive simulations that by sub-sampling a CRW, autocorrelation between subsequent turning angles can artificially be introduced, which therefore causes the sampled RW to no longer be a strict CRW; although in practice movement data with some level of autocorrelation is still often treated and analysed as a CRW. In a related but subtly different approach, Schlägel and Lewis (2016) focussed on identifying movement models that were ‘robust’ under temporal sampling, allowing specific models to be fitted to data at varying temporal resolutions, where robust was defined by specific mathematical properties of the movement model’s characteristic functions (see section 3.1 in Schlägel and Lewis (2016)). However, their work was limited to models with uniform turning angle distribution only.

Here we extend this previous work by determining the form of the probability density function (PDF) for the turning angles of the sub-sampled CRW. Our method is not limited to any specific turning angle distributions (e.g. wrapped Cauchy, wrapped normal, von Mises, SWS distributions (Jammalamadaka and Sengupta 2001)), rather it is valid for all symmetric circular distributions.

Following providing the necessary preliminaries in Sect. 2, in Sect. 3 we present the main results of this work. We provide a precise analytical expression for the turning angle and step-length distributions when a CRW is sampled at every other point. We continue to provide a method that provides the distribution of the turning angles for any sampling rate of the form $2^k$, where *k* is a positive integer, up to very close approximation. The approach used here gives a geometric interpretation of a cause for the autocorrelation observed by Nams (2013). In Sect. 4, we provide examples for where the approach of comparing the known and observed sub-sampled distributions of turning angles can be used in the analysis of movement data. Finally, in Sect. 5 we discuss the contributions of the paper and present conjectures about the precise connection between the distributions of turning angles from the sub-sampled CRW trajectories, which is an area of interest for future work.

## Background

Consider a 2-dimension discrete time, continuous space random walk (RW) in the plane, given by $\left( X\right) _{n}=\{X_{1},X_{2},\ldots ,X_{n}\}$ where $X_{t}=(x_{t},y_{t})$ denotes the location of the RW at time step *t*. Successive locations are given by $X_{t+1}=X_t+(\Delta x_t, \Delta y_t)$, where $\Delta x_t, \Delta y_t$ are the changes in the *x* and *y* coordinate respectively at time t+1, therefore giving $\Delta x_t= x_{t+1}-x_t$ and $\Delta y_t= y_{t+1}-y_t$.

Any such RW can be considered as a step-turn sequence, where the location of successive steps is given by a step-length, ℓ, equivalent to the distance between successive points, and a turning angle, θ, which denotes the angle between the previous direction of movement and the new direction. Where

1 $$\begin{aligned} \ell _{t}=\Vert \overrightarrow{X_{t}X_{t+1}}\Vert =\sqrt{(x_{t+1}-x_{t})^{2}+\left( y_{t+1}-y_{t}\right) ^{2}}=\sqrt{\Delta x_t^2+\Delta y_t^2} \end{aligned}$$

and

2 $$\begin{aligned} \theta _{X_t}= \angle \left( \overrightarrow{X_{t-1}X_{t}},\overrightarrow{X_{t}X_{t+1}}\right) \Rightarrow \cos \theta _{X_t}=\frac{\overrightarrow{X_{t-1}X_{t}}\cdot \overrightarrow{X_{t}X_{t+1}}}{\Vert \overrightarrow{X_{t-1}X_{t}}\Vert \Vert \overrightarrow{X_{t}X_{t+1}}\Vert } \end{aligned}$$

for $1\le t \le n-1$, with $(x_0,y_0), (x_1,y_1)$ given a priori. Therefore, in this way a RW can be described by the sequence $(\ell _t,\theta _{X_t})$ with step-lengths modelled by some distribution on the positive reals, $\ell _t\sim \Lambda$, and turning angles modelled by a distribution on the unit circle $\theta _t\sim f_\circ$, i.e. $f_\circ$ has domain $(-\pi ,\pi ]$. (See Appendix A for more information on these distributions.)

The simplest form of a discrete time RW is the simple random walk (SRW), which is closely related to Brownian motion. In a SRW the changes in the *x* and *y* coordinates at each time point are independently and identically drawn (i.i.d) from the normal distribution, $\Delta x, \Delta y \sim N(0,\sigma ^2)$. This leads to the equivalent step-turn process having turning angles i.i.d from the uniform distribution, $\theta _{t}\sim \text {Unif}(-\pi ,\pi )$, and step-lengths also being i.i.d, following the Rayleigh distribution, $\ell _{t}\sim R(l;\sigma ^2)$, which has mean, $\textrm{E}[\ell ]=\frac{\sigma \sqrt{2\pi }}{2}$, and variance, $\textrm{Var}[\ell ]=2\sigma ^2\left( 1-\frac{\pi }{4}\right)$ (Petrovskii et al. 2014; Ahmed et al. 2021).

**Fig. 1:**
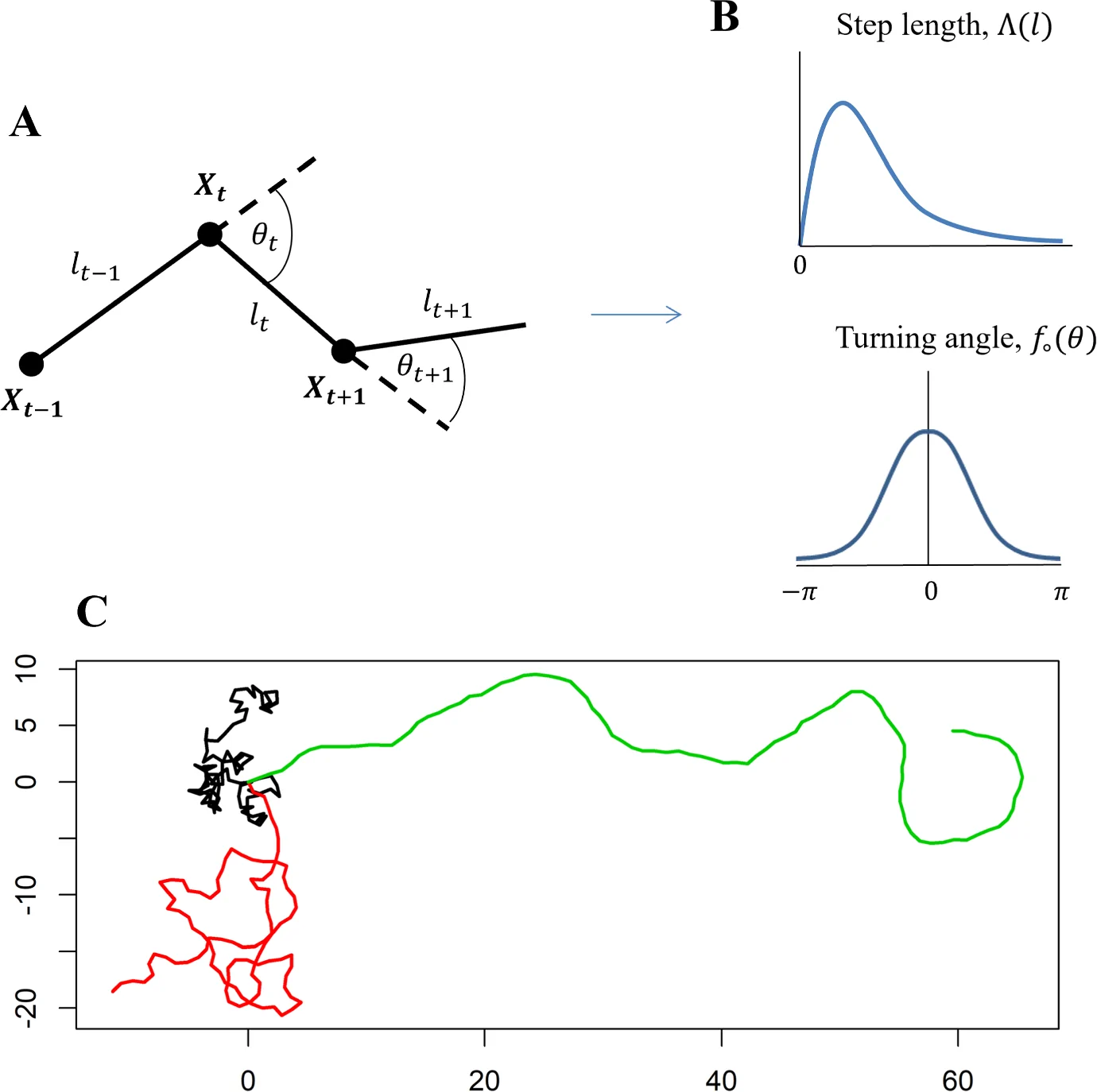
**(A)** shows the step-turn description of a discrete movement path featuring step-lengths, ℓ, and turning angles, θ, and locations *X*. **(B)** depicts the resulting step-length and turning angle distributions. **(C)** shows three CRWs formed using a constant step-length distribution and turning angles drawn from a wrapped normal (WN) distribution, each trajectory was formed with a different value for the concentration parameter for the WN distribution, ρ, with: black - ρ=0, equivalent to random movement; red - ρ=0.7; green - ρ=0.95. Figure adapted from Bailey (2023)

In a correlated random walk (CRW), in which direction of movement is expected to persist between proceeding steps, the bearings between successive steps (angle between steps taken from due North, x-axis etc.) are correlated, with successive steps locally biased towards the same direction, however the turning-angles are not correlated. A standard approach assumes the turning angles are described by a symmetric, uni-modal distribution with mean value 0, such as the wrapped Cauchy, wrapped normal or von Mises distributions (see Appendix A for more information on circular distributions). The relative ‘straightness’ of a CRW is governed by how peaked the turning angle distribution is around the value 0, which is described by the length of the mean resultant vector, ρ, calculated as the root of the sum of the squares of the distribution’s first trigonometric moments (Appendix A). Values close to 1 give near straight-line movement, with values close to 0 giving more random motion (note ρ=0 is equivalent to the circular uniform distribution where all angles in $(-\pi ,\pi ]$ are equally likely) (Fig. 1). It is also often assumed in a CRW that each step-length, $\ell _i$, and each turning angle, $\theta _{X_i}$, are i.i.d with step-lengths and turning-angles neither jointly- nor auto-correlated, which we shall assume throughout.

**Fig. 2:**
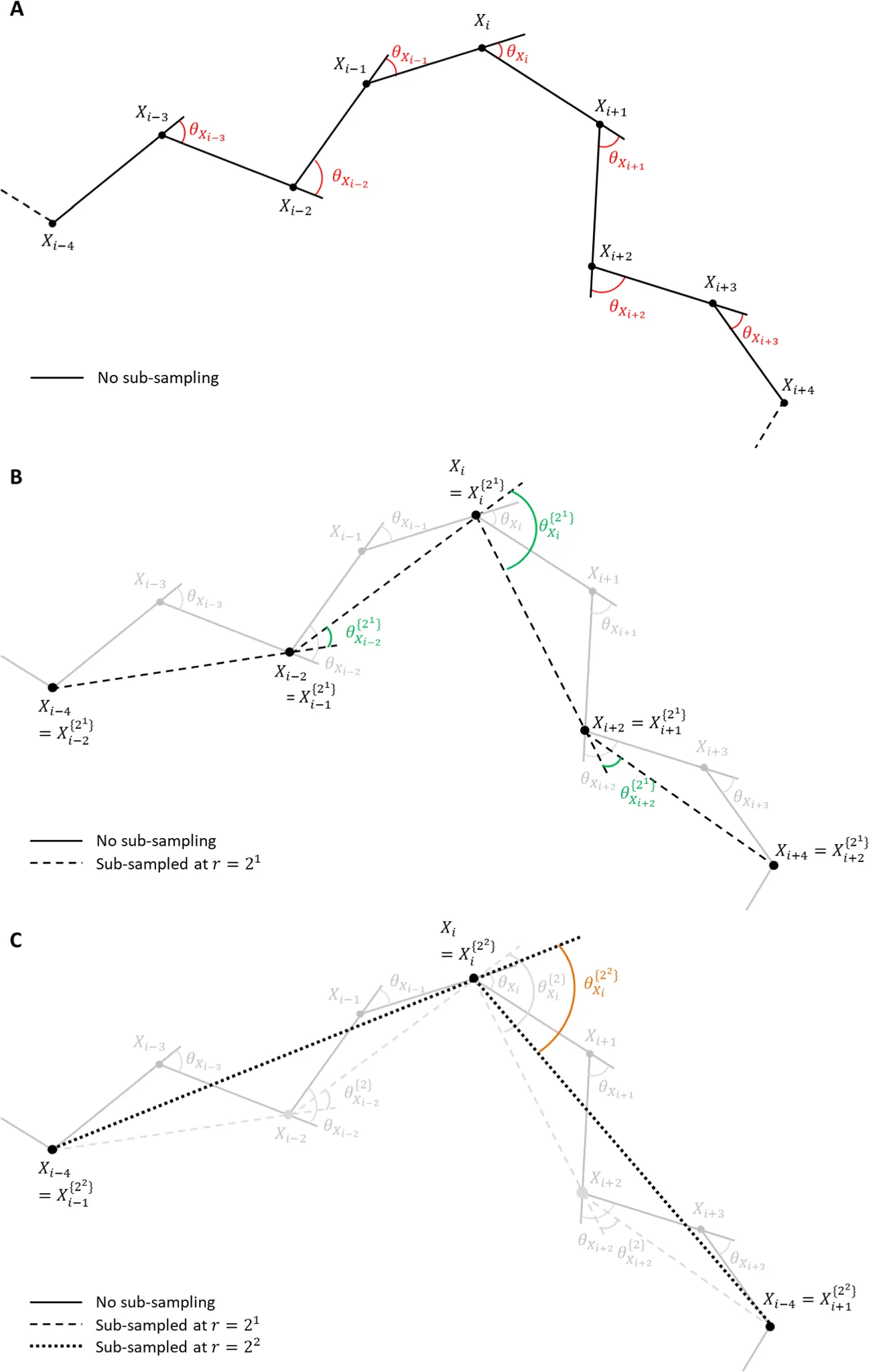
Examples of sub-sampling a RW, highlighting the resulting turning angles at each level of sampling. **(A)** is the initial, unsampled RW; **(B)** shows the initial RW sub-sampled at rate $r=2^1$; **(C)** shows the initial RW sub-sampled at rate $r=2^2$

Sub-sampling a RW is found by choosing a subset of the original locations in the initial RW, usually those which are a set number of time-steps apart (e.g. every *r*th point along the RW). Such a sub-sampled RW can also be described by using a step-turn description. Let $(X)_n$ be a CRW with turning angles, $\theta _{X_t}$, drawn from the circular distribution $f_\circ$ and step-lengths, $\ell _t$, drawn from a distribution on the positive reals Λ. Consider the sequence $\left( X\right) ^{\left\{ r\right\} }$ formed by sub-sampling the CRW given by $\left( X\right) _{n}$ at every *r*th point (Fig. 2), that is $\left( X\right) ^{\left\{ r\right\} }=\left\{ X^{\{r\}}_{1},X^{\{r\}}_{2},\ldots ,X^{\{r\}}_{t},\ldots \right\} =\left\{ X_{1},X_{1+r},\ldots ,X_{1+tr},\ldots \right\}$. This has step-turn description given by $\left( \ell ^{\{r\}}_i,\theta ^{\{r\}}_{X_i}\right)$ where,

3 $$\begin{aligned} \ell ^{\{r\}}_{i}=\Vert \overrightarrow{X^{\{r\}}_{i}X^{\{r\}}_{i+1}}\Vert =\Vert \overrightarrow{X_{i}X_{i+r}}\Vert =\sqrt{(x_{i+r}-x_{i})^{2}+\left( y_{i+r}-y_{i}\right) ^{2}} \end{aligned}$$

and

4 $$\begin{aligned} \theta ^{\{r\}}_{X_i}= &  \angle \left( \overrightarrow{X^{\{r\}}_{i-1}X^{\{r\}}_{i}},\overrightarrow{X_{i}^{\{r\}}X^{\{r\}}_{i+1}}\right) \nonumber \\= &  \angle \left( \overrightarrow{X_{i-r}X_{i}},\overrightarrow{X_{i}X_{i+r}}\right) \Rightarrow \cos \theta _{X_i}=\frac{\overrightarrow{X_{i-r}X_{i}}\cdot \overrightarrow{X_{i}X_{i+r}}}{\Vert \overrightarrow{X_{i-r}X_{i}}\Vert \Vert \overrightarrow{X_{i}X_{i+r}}\Vert } \end{aligned}$$

with $$\theta ^{\{r\}}_{X_i}\sim f_\circ ^{\{r\}}(\theta )$$ and $$\ell ^{\{r\}}_{i}\sim \Lambda ^{\{r\}}$$.

Whilst it is well known that as the sampling rate of a CRW increases, the resulting sampled RW appears closer to that of a SRW (Kareiva and Shigesada 1983; Codling et al. 2008) and, hence, the turning angle distribution tends to the circular uniform distribution, it remains an open question to describe the connection between the distribution of the initial turning angles, $f_\circ (\theta )$, with the distribution of the turning angles sampled at every *r*th step, $f_\circ ^{\{r\}}(\theta )$, and similar for the step lengths distributions $\Lambda (\ell )$ and $\Lambda ^{\{r\}}(\ell )$.

## Main claim

Here we introduce the main claims of the work. First, we consider a CRW with step-lengths of a fixed size, which will be sub-sampled at rate r=2, giving precise analytical solutions for the resulting turning angle and step-length distributions of the sub-sampled process. We then move to the more general case, looking at a CRW sampled at rate $r=2^{k}$, where $k\in \mathbb {Z}_{\ge 2}$.

First we introduce the following Remark on scaling even distribution functions:

### Remark 1

*(Scaling of an even distribution)* Let *X* be a continuous random variable with probability density function $f_X$ supported on $[X_{\min },X_{\max }]$. For any $a\in \mathbb {R}\setminus \{0\}$, the scaled random variable Y=aX has density

$$\begin{aligned} f_Y(y)=\frac{1}{|a|}f_X\!\left( \frac{y}{a}\right) , \end{aligned}$$

with support

$$\begin{aligned} \left[ \min \{aX_{\min },aX_{\max }\}, \max \{aX_{\min },aX_{\max }\} \right] , \end{aligned}$$

where by definition $f_Y(y)=0$ when *y* is not in the support. Furthermore, if $f_X$ is an even function, i.e. $f_X(x)=f_X(-x)$ for all *x*, then

$$\begin{aligned} f_Y(y) = \frac{1}{|a|}f_X\!\left( \frac{y}{|a|}\right) , \end{aligned}$$

so the distribution of *aX* depends only on |*a*|.

### Case for sampling rate r=2, with initial CRW having step-lengths of fixed size

#### Theorem 1

Let $(X)_n=\{X_0, X_1, X_2,\ldots ,X_n\}$ be a correlated random walk in $\mathbb {R}^2$ with step-turn characterisation $(\ell _i,\theta _{X_t})$, where $\ell _{t}=\ell \in \mathbb {R}_{>0}$, a fixed positive real value for all *t*, and $\theta _{X_t}\sim f_{\circ }$ where $f_{\circ }$ is a symmetric circular distribution centred around 0. The subsequent RW formed by sub-sampling $(X)_n$ at rate r=2, has circular turning-angle distribution $f^{\{2\}}_{\circ }$ given by

$$\begin{aligned} f^{\{2\}}_{\circ }(\theta )=\sum _{m=-1}^{1}f^{\{2\}}(\theta +2m\pi ), \end{aligned}$$

where

5 $$\begin{aligned} f^{\{2\}}(\theta )=4f_{\circ }(\theta )*f^{*2}_{\circ }(2\theta ), \end{aligned}$$

and $f^{*2}$ is the power convolution of *f* (i.e. $f^{*2}{\mathop {=}\limits ^{\text {def}}}f*f$).

#### Proof

Let $(X)_{n}$ be a correlated random walk as in Theorem 1. For a sampling rate of r=2, every other point of $(X)_{n}$ will be chosen to form $(X)^{\{2\}}$ which will have step lengths $\ell ^{\{2\}}_{t}$ drawn from some distribution $\Lambda ^{\{2\}}$ and turning angles $\theta ^{\{2\}}_{X_t}$ drawn from some distribution $f^{\{2\}}_{\circ }(\theta )$.

**Fig. 3:**
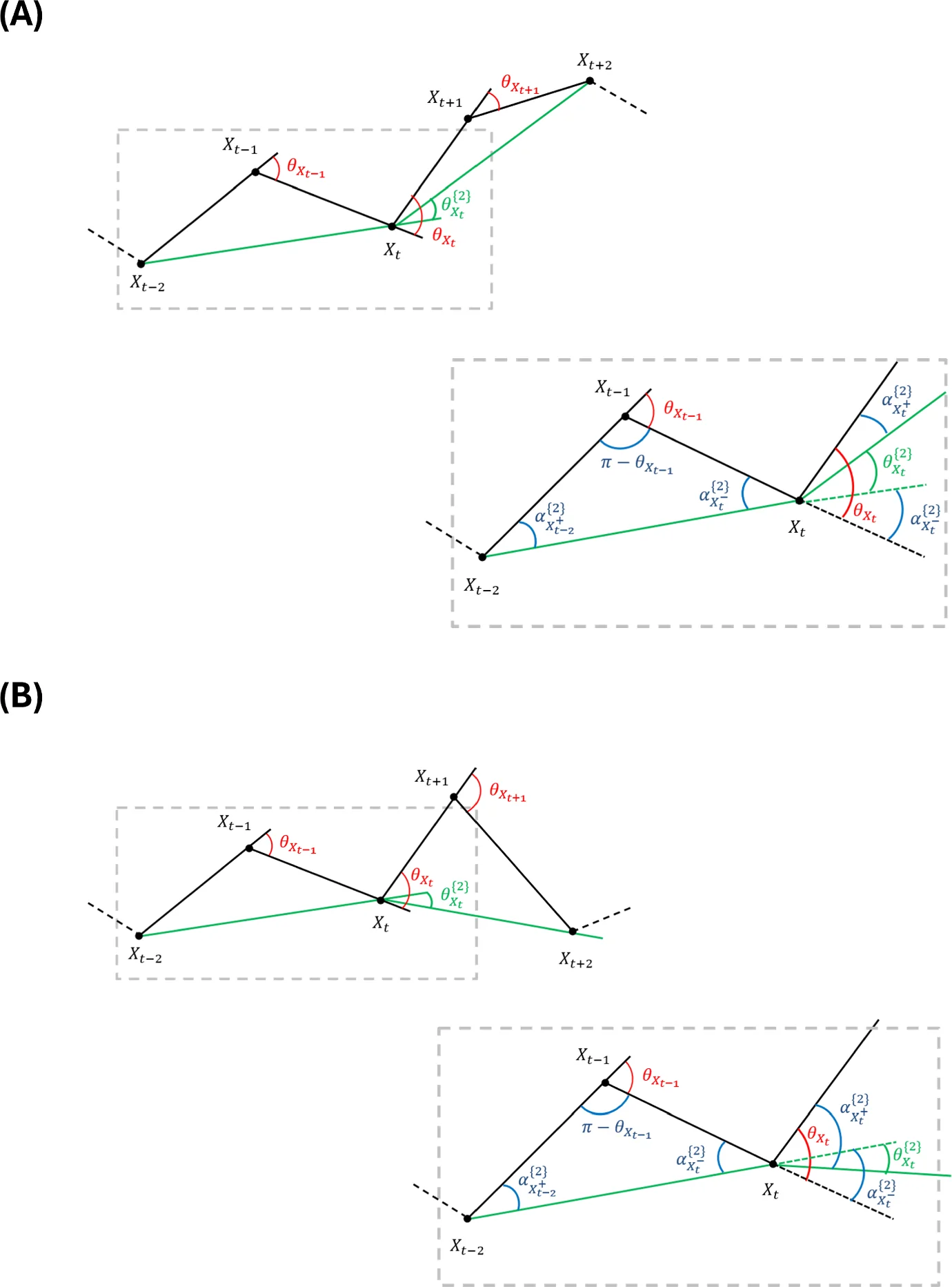
Figure depicting the triangle $T_t=\triangle X_t,X_{t-1},X_{t-2}$, for two different realisations of a sub-sampled RW, demonstrating how the geometry of the RW affects whether $\sigma _i$ returns +1 or -1. Both figures feature a similar RW, differing only by the location of $X_{t+2}$. In **(A)** the expression for $\theta _{X_t}$ would be given by $\alpha ^{\{2\}}_{X^+_t}+\alpha ^{\{2\}}_{X^-_t}+\theta ^{\{2\}}_{X_t}$, equivalent to σ=+1 in all three terms, whereas, in **(B)** the expression for $\theta _{X_t}$ is now given by $\alpha ^{\{2\}}_{X^+_t}+\alpha ^{\{2\}}_{X^-_t}-\theta ^{\{2\}}_{X_t}$, equivalent to $\sigma _{\alpha }=+1$ and $\sigma _{\theta }=-1$. This is due to the overlap of angle $\theta ^{\{2\}}_{X_t}$ with angles $\alpha _{X_t^+}$ and $\alpha _{X_t^-}$, whereas in **A** there was no such overlap

Consider the point $X_t$, which w.l.o.g. we can assume is in both the initial and the sub-sampled walks. The angle $\theta ^{\{2\}}_{X_t}$ is therefore the angle between movement directions $\overrightarrow{X_{t-2}X_t}$ and $\overrightarrow{X_{t}X_{t+2}}$ (see Figs 2 and 3). Now let $T_t$ be the triangle formed by the point $X_t$ and the two previous locations (i.e. $T_t=\bigtriangleup X_tX_{t-1}X_{t-2}$). This must be isosceles as the distances between successive points, are constant ($||X_{t-2}, X_{t-1}||=||X_{t-1}, X_{t}||=\ell$). Denote the internal angle of this triangle at $X_t$ as $\alpha ^{\{2\}}_{X^-_t}$, that is $\alpha ^{\{2\}}_{X^-_t}=\angle \left( X_{t-2}X_tX_{t-1}\right)$, where the $\{-\}$ in the subscript denotes that this is the internal angle formed from the *prior* two locations at $X_t$. Similarly, we denote the internal angle of the triangle $T_t$ at $X_{t-2}$ as $\alpha ^{\{2\}}_{X^+_{t-2}}$ where the $\{+\}$ denotes that this is the internal angle at $X_{t-2}$ and the *succeeding* two points (Fig. 3). As $T_t$ is isosceles the internal angle at $X_{t-2}$ must equal the internal angle at $X_{t}$, hence $\alpha ^{\{2\}}_{X^+_{t-2}}=\alpha ^{\{2\}}_{X^-_{t}}$. The remaining internal angle at $X_{t-1}$ is simply $\left( \pi -\theta _{X_{t-1}}\right)$, therefore we have

6 $$\begin{aligned} \alpha ^{\{2\}}_{X^+_{t-2}}+\alpha ^{\{2\}}_{X^-_t}+(\pi -\theta _{X_{t-1}})&= \pi \nonumber \\ \Rightarrow \alpha ^{\{2\}}_{X^-_t}+\alpha ^{\{2\}}_{X^-_t}+(\pi -\theta _{X_{t-1}})&= \pi ,\nonumber \\ \text {hence,} \quad 2\alpha ^{\{2\}}_{X^-_t}&= \theta _{X_{t-1}}\nonumber \\ \Rightarrow \alpha ^{\{2\}}_{X^-_t}&=\frac{1}{2}\theta _{X_{t-1}} \end{aligned}$$

Repeating this approach for the triangle formed by $X_t$ and the two subsequent locations, $X_{t+1}, X_{t+2}$, gives

7 $$\begin{aligned} \alpha ^{\{2\}}_{X^+_t}=\frac{1}{2}\theta _{X_{t+1}}, \end{aligned}$$

where $\alpha ^{\{2\}}_{X^+_t}=\angle \left( X_{t+1}X_{t}X_{t+2}\right)$. From Fig. 3 and Eqs. 6–7, we have

8 $$\begin{aligned} \nonumber \theta ^{\{2\}}_{X_t}&=\sigma _{(1)}\theta _{X_t}+\sigma _{(2)}\alpha ^{\{2\}}_{X^-_t}+\sigma _{(3)}\alpha ^{\{2\}}_{X^+_t}\\&=\sigma _{(1)}\theta _{X_t} +\frac{\sigma _{(2)}}{2}\theta _{X_{t-1}} +\frac{\sigma _{(3)}}{2}\theta _{X_{t+1}}, \end{aligned}$$

where each $\sigma _{(i)}\in \{-1,1\}$ is determined by the local geometry of the random walk (Fig. 3).

Since $\theta _{X_t}$, $\theta _{X_{t-1}}$ and $\theta _{X_{t+1}}$ are independent and identically distributed with density $f_\circ$, the distribution of $\theta ^{\{2\}}_{X_t}$ is obtained by convolving the distributions of the three terms in Eq. 8. Furthermore, as $f_\circ$ is symmetric about zero, Remark 1 implies that the distributions of $\sigma _{(i)}\theta$ and θ are identical for $\sigma _{(i)}\in \{-1,1\}$. Consequently, the values of the $\sigma _{(i)}$ in Eq. 8 do not affect the resulting convolution, giving

9 $$\begin{aligned} \nonumber f^{\{2\}}(\theta )&= f_\circ (\theta )*2f_\circ (2\theta )*2f_\circ (2\theta )\\&= 4f_\circ (\theta )*f_\circ ^{*2}(2\theta ). \end{aligned}$$

where $f^{*2}_{\circ }(2\theta ){\mathop {=}\limits ^{def}}f_{\circ }(2\theta )*f_{\circ }(2\theta )$. By Remark 1, with a=2, $f_\circ (2\theta )$ has support $[-\pi /2,\pi /2]$, and therefore $f^{\{2\}}(\theta )$ has support $[-2\pi ,2\pi ]$. Finally, we transform $f^{\{2\}}(\theta )$ into a circular distribution with domain $\theta \in [-\pi ,\pi ]$ by wrapping around the unit circle and denoting as $f^{\{2\}}_{\circ }$, to give

10 $$\begin{aligned} f^{\{2\}}_{\circ }(\theta )=\sum _{m=-1}^{1}f^{\{2\}}(\theta +2m\pi ). \end{aligned}$$

□

**Fig. 4:**
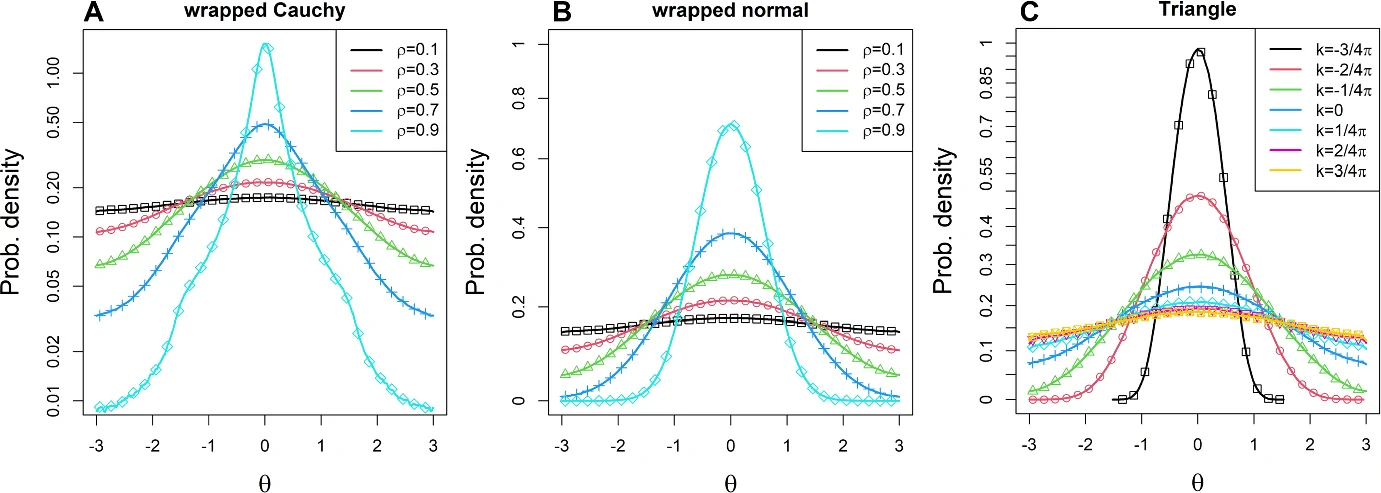
Comparison of predicted and simulated turning-angle distributions for CRWs sub-sampled at rate r=2. Points show the predicted values from Theorem 1 (evaluated at $\theta \in [-\pi ,\pi ]$ in increments of 0.2), and solid lines show the corresponding simulated PDFs. Panels: **(A)** wrapped Cauchy; **(B)** wrapped normal; **(C)** wrapped triangle. For each distribution, concentration parameters vary as indicated in the legend. The simulated distributions were found by sub-sampling a CRW consisting of $10^8$ unit length steps with turning angles drawn from a zero-centred circular distribution. The predicted PDFs were obtained by repeated evaluation of Eq. 8, where for each term, $10^8$ random angles were drawn, summed modulo 2π, and used to construct $f_\circ ^{\{2\}}$. All computations were performed in *R*. The *y*-axis is log-transformed for visual clarity

To demonstrate Theorem 1, the turning-angle PDFs were compared with those obtained from repeated simulations of sub-sampled CRWs. Figure 4 displays these comparisons across several circular distributions and concentration parameters. In all cases, the simulated PDFs match the theoretical PDFs obtained from Theorem 1, demonstrating the validity of the theorem.

#### Step-length distribution for sub-sampled RW from Theorem 1

As a consequence of determining the turning angle distribution $f^{\{2\}}_{\circ }$, the step-length distribution $\Lambda ^{\{2\}}$, can also be precisely calculated:

##### Corollary 1

For a correlated random walk with constant step-length distribution, that is, for all ℓ we have $\Lambda (\ell )=\ell ^*\in \mathbb {R}$, and turning angles, $\theta _t$, drawn from a circular distribution $f_\circ$ centred and symmetric around 0, the distribution of step-lengths of the random walk formed by sub-sampling at rate r=2 is given by

11 $$\begin{aligned} \Lambda ^{\{2\}}(\ell ^{\{2\}})=\frac{2}{\sqrt{(\ell ^*)^2-\left( \frac{\ell ^{\{2\}}}{2}\right) ^2}}f_\circ \left( 2\arccos {\left( \frac{\ell ^{\{2\}}}{2\ell ^*}\right) }\right) \end{aligned}$$

which has range $[0,2\ell ^*]$.

##### Proof

Consider the consecutive points of a CRW $X_{t-1},X_t,X_{t+1}$. We have $|X_{t-1}X_t|=|X_tX_{t+1}|=\ell ^*$ by the properties of Λ, we are interested in describing the distribution of $\ell _t^{\{2\}}=|X_{t-1}X_{t+1}|$. Using the cosine rule, The length of the vector between points $X_{t-1}$ and $X_{t+1}$ for all *t* is simply

12 $$\begin{aligned} \nonumber |X_{t-1}X_{t+1}|^2&=|X_{t-1}X_t|^2+|X_{t}X_{t+1}|^2-2|X_{t-1}X_t||X_{t}X_{t+1}|\cos {\left( \pi -\theta _{X_t}\right) }\\ \nonumber \left( \ell _t^{\{2\}}\right) ^2&=2\ell ^{*2}+2\ell ^{*2}\cos {\left( \theta _{X_t}\right) }\\ \Rightarrow \ell _t^{\{2\}}&=\sqrt{2\ell ^{*2}(1+\cos {\left( \theta _{X_t}\right) })}=2\ell ^*\cos {\frac{\theta _{X_t}}{2}} \end{aligned}$$

This shows that the distribution of $\ell ^{\{2\}}$ is given in terms of the distribution of θ, with $\ell _t^{\{2\}}$ taking values in $[0,2\ell ^*]$ as $\theta _{X_t}\in (-\pi ,\pi ]$. Now, as all $\theta _{X_t}$ are i.i.d, to find the distribution, $\Lambda ^{\{2\}}(\ell ^{\{2\}})$, we have

13 $$\begin{aligned} \nonumber F_{\Lambda ^{\{2\}}}(\ell ^{\{2\}})&=P(L\le \ell ^{\{2\}})\\\nonumber&=P\left( 2\ell ^*\cos {\frac{\theta }{2}}\le \ell ^{\{2\}}\right) \\\nonumber&=2P\left( |\theta |\ge 2\arccos {\left( \frac{\ell ^{\{2\}})}{2\ell ^*}\right) }\right) \\&=2\left[ 1-F_\theta \left( 2\arccos {\left( \frac{\ell ^{\{2\}})}{2\ell ^*}\right) }\right) \right] \end{aligned}$$

where $F_{\Lambda ^{\{2\}}}, F_\theta$ are the CDFs of $\Lambda ^{\{2\}}$ and $f_\circ$ respectively. The coefficient of 2 appears due to θ having domain $[-\pi ,\pi ]$, arccos having the restricted range of [0,π] and cosine being an even function. Hence, the distribution $\Lambda ^{\{2\}}$ is given by

14 $$\begin{aligned} \nonumber \Lambda ^{\{2\}}(\ell ^{\{2\}})=\frac{dF_{\Lambda ^{\{2\}}}(\ell ^{\{2\}})}{d\ell ^{\{2\}}}&=2\frac{dF_\theta }{d\theta }\Bigg |\frac{d\theta }{d\ell ^{\{2\}}}\Bigg |\\ \nonumber&=2\frac{dF_\theta }{d\theta }\Bigg |\frac{d}{d\ell ^{\{2\}}}\left( 2\arccos {\left( \frac{\ell ^{\{2\}}}{2\ell ^*}\right) }\right) \Bigg |\\&=\frac{2}{\sqrt{\ell ^{*2}-\left( \frac{\ell ^{\{2\}}}{2}\right) ^2}}f_\circ \left( 2\arccos {\left( \frac{\ell ^{\{2\}}}{2\ell ^*}\right) }\right) , \end{aligned}$$

Noting that here we have $\frac{dF_\theta }{d\theta }=f_\circ (\theta )$ and $\theta =2\arccos {\left( \frac{\ell ^{\{2\}})}{2\ell ^*}\right) }$. □

**Fig. 5:**
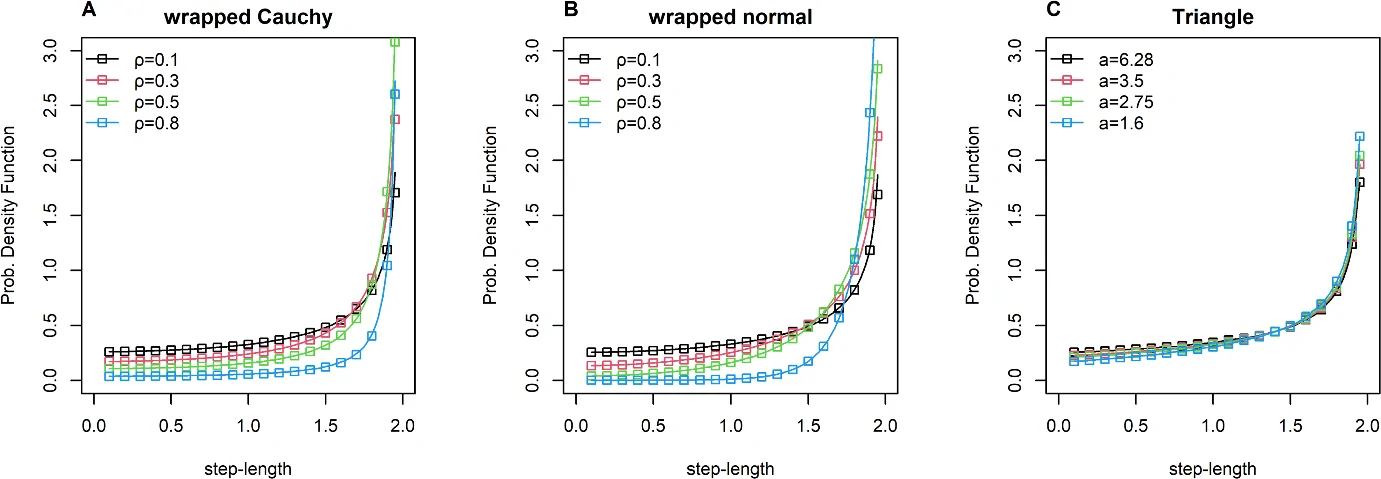
Comparison of predicted and simulated step-length distributions for CRWs with fixed step length $\ell ^*=1$ sub-sampled at rate r=2. Turning angles were drawn from zero-centred wrapped Cauchy (**A**), wrapped normal (**B**), and wrapped triangle (**C**) distributions. In panels (A) and (B), black, red, green, and blue curves correspond to mean resultant vectors ρ=0.1,0.3,0.5,0.8. In panel (**C**), parameter values a=6.28,3.5,2.75,1.6 (black, red, green, blue) were chosen to approximate the same MRV values (see Appendix A). Simulated results (solid lines) were obtained by measuring the step-lengths along a sub-sampled CRW generated from an initial $10^8$ unit length steps. A smooth, non-parametric estimate of the empirical distribution was then produced using the ‘density()’ function in ‘*R*’, which fits a continuous curve to the raw simulated data. Predicted step-length distributions (points) were computed using Eq. 12 from Corollary 1. All computations were performed in ‘*R*’

To demonstrate Corollary 1, the analytical expression for $\Lambda ^{\{2\}}$ was compared with distribution of step-lengths obtained from simulated sub-sampled CRWs. Figure 5 shows that the theoretical predictions agree with the simulated distributions across a range of circular distributions and concentration parameters, demonstrating the validity of Corollary 1.

### Case for sampling rate of $r=2^k$ where k≥2

We now look to describe the distribution of the turning angles in the RW formed by sub-sampling at a rate of $r=2^k$, for integers k≥2. We will do this by describing the turning angle $\theta _{X_t}^{\{2^k\}}$ in a similar method to that used for $\theta _{X_t}^{\{2\}}$ in the proof of Theorem 1. We then build the angles from the sampled RW $\theta _{X_t}^{\{r\}}$ as a linear sum of angles from the initial unsampled RW so as to be of the form, $\theta _{X_t}^{\{r\}}=\sum _\tau a_{t+\tau }\theta _{X_{t+\tau }}$, where $a_{t+\tau }$ are constants. In this linear sum, each $\theta _{X_{t+\tau }}$ appears once and as these are i.i.d with distribution $f_{\circ }(\theta )$, we find the distribution of $\theta _{X_t}^{\{r\}}$ by convoluting the distributions of the $a_{t+\tau }\theta _{X_{t+\tau }}$ terms.

The proof proceeds in four stages. First, we derive a recursive representation for the turning angles. Second, we approximate the recursion by replacing the unknown signs of each term with their expectations. Third, we solve the resulting coefficient recursion. Finally, we obtain the turning-angle density by convolution and wrap the result onto the unit circle.

We begin by introducing the notation we will use for the remainder of the work:

- $(X)^{\{2^{k}\}}$ is the RW formed by sub-sampling the RW $(X)_n$ with sampling rate $r=2^{k}$.
- $\theta ^{\{2^k\}}_{X_t}$ is the turning angle at point $X_t$ for the sub-sampled RW $(X)^{\{2^{k}\}}$. i.e. the angle between $\overrightarrow{X_{t-2^k}X_t}$ and $\overrightarrow{X_tX_{t+2^k}}$.
- $f_{\circ }^{\{2^{k}\}}$ is the circular distribution of the turning angles, $\theta _{X_t}^{\{2^k\}}$, for the sub-sampled random walk with rate $2^k$.
- The initial turning angle distribution for the un-sampled random walk (r=1⇔k=0) is given by $f_{\circ }^{\{1\}}$, however, for sake of ease we drop the superscripts in this case. i.e. $\theta _{X_t}^{\{1\}}=\theta _{X_t}$ and $f_{\circ }^{\{1\}}=f_{\circ }$.
- $\alpha ^{\{2^{k}\}}_{X^-_t}$, is the internal angle of the triangle formed at point $X_t$ and the *previous* two locations of $X_{t-2^{k-1}}, X_{t-2^{k}}$; that is,
  $$\begin{aligned} \alpha ^{\{2^{k}\}}_{X^-_t}=\angle \left( X_{t-2^k},X_t, X_{t-2^{k-1}}\right) . \end{aligned}$$
- $\alpha ^{\{2^{k}\}}_{X^+_t}$, is the internal angle of the triangle formed at point $X_t$ and the *subsequent* two locations of $X_{t+2^{k-1}}, X_{t+2^{k}}$; that is,
  $$\begin{aligned} \alpha ^{\{2^{k}\}}_{X^+_t}=\angle \left( X_{t+2^k},X_t, X_{t+2^{k-1}}\right) . \end{aligned}$$
- $T^{\{2^{k}\}}_t$ is the triangle formed by points $X_t$ and the previous two points in the random walk sub-sampled at rate $r=2^{k-1}$ i.e the triangle $\triangle X_t, X_{t-2^{k-1}}, X_{t-2^{k}}$. See Fig. 3 for the case k=1.
- Following Theorem 1 and Fig. 2, the turning angle, $\theta ^{\{2^{k}\}}_{X_t}$, in the sub-sampled RW at location $X_t$ is given by
  15 $$\begin{aligned} \theta ^{\{2^{k}\}}_{X_t}=\sigma _{(k,1)}\theta ^{\{2^{k-1}\}}_{X_t}+\sigma _{(k,2)}\alpha ^{\{2^{k}\}}_{X^-_t}+\sigma _{(k,3)}\alpha ^{\{2^{k}\}}_{X^+_t}, \end{aligned}$$
  where $\sigma _{(k,i)}\in \{-1,1\}$ denotes the sign for the contribution of each term in the expression, with *k* denoting the sampling rate and *i* indexing the location of terms in Eqn. 15. As discussed in the proof of Theorem 1, the precise value of $\sigma _{(k,i)}$ is governed by the local geometry of the underlying random walk (see Fig. 3).

**Assumptions and approximations**

To obtain an analytically tractable expression for the turning angle distribution of the sub-sampled RW at rate $r=2^k, k\ge 2$, we make two approximations.

(i) We define $\Lambda _\triangle (\ell )$ to be a distribution on the positive reals such that if a CRW has step length distribution $\Lambda _\triangle (\ell )$ then the triangles $T^{\{2^{k}\}}_t$ are approximately isosceles with $\angle X_{t-2^{k-1}}X_{t-2^k},X_t=\angle X_{t-2^{k-1}}X_{t},X_{t-2^k}$ for all *t*. That is, when the step length distribution is $\Lambda _\triangle (\ell )$, the triangulation technique deployed in the proof of Theorem 1 may be assumed to hold for all sampling rates. Simulations demonstrate this is a reasonable assumption for ‘standard’ step length distributions (see Appendix C) and therefore in Theorem 2 we assume the step length distribution is one such $\Lambda _\triangle (\ell )$.
(ii) During sub-sampling, the local geometry of a RW becomes intractably complex, we therefore model $\sigma _{(k,i)}$ as generalized Rademacher random variables,
  $$\begin{aligned} \sigma _{(k,i)}\sim \text {Rad}\left( p(k,i)\right) \Leftrightarrow \sigma _{(k,i)}={\left\{ \begin{array}{ll}+1, \quad \text {with probability }p(k,i)\\ -1, \quad \text {with probability }1-p(k,i), \end{array}\right. } \end{aligned}$$
  where *p*(*k*, *i*) is a function of the sampling rate *k* and the indexing term, *i*, as described in Eqn. 15. It is further assumed that *p*(*k*, *i*) is constant at all $X_t$, all sampling rates, and all indexing terms *i*, therefore giving p(k,i)=p for all *k* and *i*, and hence,
  $$\begin{aligned} \sigma _{(k,i)}=\sigma \sim \text {Rad}(p). \end{aligned}$$

The following theorem is derived under these assumptions.

#### Theorem 2

Consider a discrete time CRW in $\mathbb {R}^2$, defined by turning angles, $\theta _t$, independently drawn from $f_\circ (\theta )$, a zero-centred, symmetric circular distribution and step-lengths drawn independently from $\Lambda _\triangle (\ell )$, a distribution on the positive reals satisfying the triangulation property described above. further that the signs arising from the local geometry are modelled as independent generalized Rademacher variables with $\Pr (\sigma _{(k,i)}=1)=p_{k,i}=p$ at every sub-sampling level *k*. Then the turning angle distribution of the sub-sampled RW at rate $r=2^k, k\ge 2$ is given by

$$\begin{aligned} f^{\{2^k\}}_{\circ }(\theta )\approx \sum _{m=-(2^k-1)}^{2^k-1}f^{\{2^k\}}(\theta +2m\pi ) \end{aligned}$$

where16with $f^{*2}$ the power convolution of *f* (i.e. $f^{*2}{\mathop {=}\limits ^{\text {def}}}f*f$), and $p\in \left( \frac{1}{2},1\right]$.

#### Proof

Let us consider a RW which has been sampled at the rate $r=2^k$ (with k≥2). At the point $X_t$, we wish to describe the turning angle $\theta ^{\{2^k\}}_{X_t}$, (the angle between $\overrightarrow{X_{t-2^k}X_t}$ and $\overrightarrow{X_tX_{t+2^k}}$). Following the arguments within the proof of Theorem 1 (see Eq. 8), we have at point $X_t$;

17 $$\begin{aligned} \theta _{X_t}^{\{2^{k}\}}=\sigma _{(k,1)}\theta _{X_t}^{\{2^{k-1}\}}+\sigma _{(k,2)}\alpha _{X^-_t}^{\{2^{k}\}}+\sigma _{(k,3)}\alpha _{X^+_t}^{\{2^{k}\}}. \end{aligned}$$

Since each $\sigma _{(k,i)}$ is modelled as a Rademacher random variable, σ with parameter *p*, we get

18 $$\begin{aligned} \theta _{X_t}^{\{2^{k}\}}=\sigma _1\theta _{X_t}^{\{2^{k-1}\}}+\sigma _2\alpha _{X^-_t}^{\{2^{k}\}}+\sigma _3\alpha _{X^+_t}^{\{2^{k}\}}. \end{aligned}$$

where $\sigma _1, \sigma _2,\sigma _3\sim \textrm{Rad}(p)$. Furthermore, using the assumption that the local triangles formed after repeated sub-sampling, $T^{\{2^k\}}_t$, may be treated as isosceles, gives

19 $$\begin{aligned} \nonumber \alpha _{X^-_t}^{\{2^{k}\}}\approx&\frac{1}{2}{\theta ^{\{2^{k-1}\}}_{X_{t-2^{k-1}}}}\\ \alpha _{X^+_t}^{\{2^{k}\}}\approx&\frac{1}{2}{\theta ^{\{2^{k-1}\}}_{X_{t+2^{k-1}}}}. \end{aligned}$$

Substituting into Eq. 18 gives

20 $$\begin{aligned} \theta _{X_t}^{\{2^{k}\}}\approx \sigma _1\theta _{X_t}^{\{2^{k-1}\}}+\sigma _2\frac{1}{2}{\theta ^{\{2^{k-1}\}}_{X_{t-2^{k-1}}}}+\sigma _3\frac{1}{2}{\theta ^{\{2^{k-1}\}}_{X_{t+2^{k-1}}}} \end{aligned}$$

Iteratively applying Eq. 20 will yield a linear expression for $\theta _{X_t}^{\{2^{k}\}}$ in terms of $\theta _{X_{t+\tau }}^{\{1\}}=\theta _{X_{t+\tau }}$. Note that *k* iterations of Eq. 20 are required to reduce the exponents in the RHS from $2^k$ to 1. As each iteration will add/subtract a term of the form $2^{k-j}$ to the subscripts of $X_t$ (see Eq. 19), (where $j=\{1,2,\ldots ,k\}$), for terms of the form $X_{t+\tau }$, the maximum value τ can be is $\tau =2^{k-1}+2^{k-2}+\cdots +2+1=2^k-1$, and by the symmetry of Eq. 20, τ will have a minimum of $t-(2^k-1)$.

We therefore have

21 $$\begin{aligned} \theta _{X_t}^{\{2^{k}\}}=\sum _{\tau =-(2^k-1)}^{2^k-1} a^{\{2^k\}}_{t+\tau } \theta _{X_{t+\tau }} \end{aligned}$$

where the coefficients $a^{\{2^k\}}_{t+\tau }$ are to be determined.

**Fig. 6:**
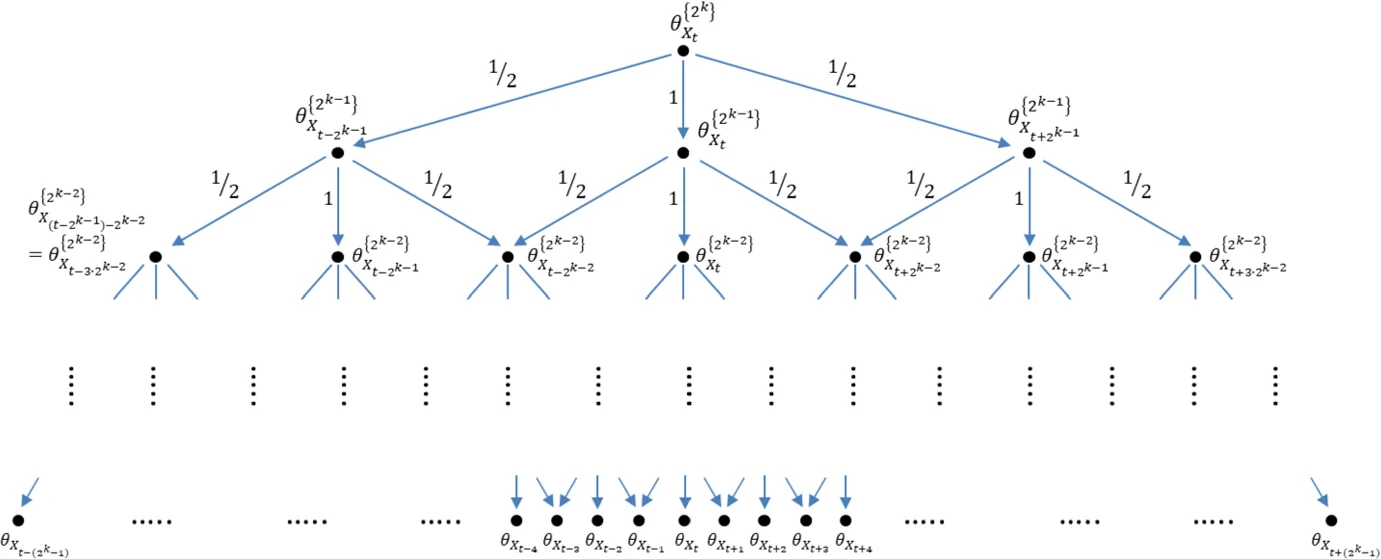
Directed graph showing the recursive relations given in Eq. 20. Summing the product of edge weights (sign dependent on probabilities $p_j$) along all paths from the root to a leaf gives the coefficient of that leaf’s label in Eq. 21

Repeated application of Eq. 20 naturally defines the recursion tree shown in Fig. 6, which provides an efficient way to determine the coefficients in Eq. 21. At level *j* of the tree (j=0 at the root and j=k at the leaves), each edge has positive sign with probability $\Pr (\sigma =+1)=p$ and negative sign with probability 1-p. The coefficient $a_{t+\tau }^{\{2^k\}}$ of a given term $\theta _{t+\tau }$ is obtained by summing the weights of all paths from the root vertex $\theta _{X_t}^{\{2^k\}}$ to the corresponding leaf. Each path weight is the product of the edge weights along that path and represents one occurrence of $\theta _{t+\tau }$ generated by repeated application of Eq. 20 (see Appendix B for an example with sampling rate $r=4=2^2$).

Rather than considering every root-to-leaf path, the coefficients can be computed recursively by exploiting the local connectivity of the tree. Let $a_{t+\tau }^j$ denote the total weight of all paths from level j-1 to the vertex $\theta _{X_{t+\tau }}^{\{2^{k-j}\}}$ at level *j*. By symmetry $a^{\{2^k\}}_{t+\tau }=a^{\{2^k\}}_{t-\tau }$, thus without loss of generality we may consider only the non-negative values of τ. These coefficients satisfy the recursion

22 $$\begin{aligned} a^{j}_{t+\tau }= {\left\{ \begin{array}{ll} \sigma a^{j-1}_{t+\frac{\tau }{2}},\quad \tau \text { even},\\ \frac{1}{2}\sigma a^{j-1}_{t+(\frac{\tau -1}{2})},\quad \tau = 2^{j}-1\\ \frac{1}{2}\left[ \sigma _1a^{j-1}_{t+\frac{\tau -1}{2}}+\sigma _2a^{{j-1}}_{t+\frac{\tau +1}{2}}\right] ,\; \text {else,} \end{array}\right. } \end{aligned}$$

for j=1,⋯,k, $\tau = 0, \dots , 2^k-1$, with $a^0_{t+\tau }=1$. Note, at the final level with j=k, the coefficients $a^{j}_{t+\tau }$ are precisely those of $a^{\{2^k\}}_{t+\tau }$ in Eq. 21. In Eq. 22, the first case corresponds to those vertices in Fig. 6 with one vertical arrow entering them (e.g. $\theta _{X_t}^{\{2^{k-2}\}}$); the second case corresponds to the extreme right/left branches with one left/right arrow entering them (e.g. $\theta _{X_{t-2^{k-1}}}^{\{2^{k-1}\}}$); and the third case is for those vertices which have two arrows entering into them (e.g. $\theta _{X_{t-2^{k-2}}}^{\{2^{k-2}\}}$). This demonstrates that for $\tau \ne 0$ the coefficient $a^{\{2^k\}}_{t+\tau }$ is a function of additions and subtractions of all reachable vertices above it, and is dependent on *p*. Taking the expectation of $a^j_{t+\tau }$ in Eq. 22 gives

23 $$\begin{aligned} \tilde{a}^{j}_{t+\tau }= \textrm{E}\left[ a^{j}_{t+\tau }\right] = {\left\{ \begin{array}{ll} (2p-1)\tilde{a}^{j-1}_{t+\frac{\tau }{2}},\quad \tau \text { even},\\ \frac{1}{2}(2p-1)\tilde{a}^{j-1}_{t+(\frac{\tau -1}{2})},\quad \tau =2^{j}-1\\ \frac{1}{2}(2p-1)\left[ \tilde{a}^{j-1}_{t+\frac{\tau -1}{2}}+\tilde{a}^{j-1}_{t+\frac{\tau +1}{2}}\right] ,\; \text {else} \end{array}\right. } \end{aligned}$$

for j=1,⋯,k, $\tau = 0, \dots , 2^k-1$, and $\tilde{a}^{0}_{t+\tau }=1$. Where we have used

24 $$\begin{aligned} \textrm{E}\left[ \sigma _i\right] =(+1)p + (-1)(1-p)=(2p-1) \end{aligned}$$

and assumed that $\left[ \sigma _1\tilde{a}^{j}_{t+\frac{\tau -1}{2}}+\sigma _2\tilde{a}^{j}_{t+\frac{\tau +1}{2}}\right]$ is linear in respect to the expectation operator.

The coefficients $\tilde{a}^{j}_{t+\tau }$, can be readily found by repeatedly applying Eq. 23 and (recalling $\tilde{a}^{j}_{t+\tau }=\tilde{a}^{j}_{t-\tau }$) can be written more compactly as

25 $$\begin{aligned} \tilde{a}^{\{2^k\}}_{t+\tau }=\frac{2^k-|\tau |}{2^k}(2p-1)^k, \end{aligned}$$

where k≥0, and $\tau =-(2^k-1),\ldots ,2^k-1$ (see Appendix D for the proof). Hence, using Eq. 21 and Eq. 25 we can write the angle at $X_t$ of the sub-sampled random walk, $\theta _{X_t}^{\{2^k\}}$, as

26 $$\begin{aligned} \theta _{X_t}^{\{2^k\}}\approx&\sum _{\tau =-(2^k-1)}^{2^k-1} \tilde{a}^{\{2^k\}}_{t+\tau } \theta _{X_{t+\tau }} \end{aligned}$$

27 $$\begin{aligned} =&\sum _{\tau =-(2^k-1)}^{2^k-1} \frac{2^k-|\tau |}{2^k}(2p-1)^k\theta _{X_{t+\tau }}\nonumber \\ =&\sum _{\tau =-(2^k-1)}^{2^k-1}(p-\frac{1}{2})^k(2^k-|\tau |)\theta _{X_{t+\tau }}. \end{aligned}$$

Hence, since $\theta _{X_{t+\tau }}$ are i.i.d, the distribution of $\theta _{X_t}^{\{2^k\}}$, can be found by a convolution of the distributions for the $\tilde{a}^{\{2^k\}}_{t+\tau } \theta _{X_{t+\tau }}$ terms.

Denoting the distribution of $\tilde{a}^{\{2^k\}}_{t+\tau } \theta _{X_{t+\tau }}$ as $f_\tau$, applying Remark 1, and noting that all $\theta _{X_{t+\tau }}$ are modelled by the distribution $f_\circ (\theta )$, gives

28 $$\begin{aligned} f_\tau (\theta )=\frac{1}{|(p-\frac{1}{2})^k||2^k-|\tau ||}f_\circ \left( \frac{\theta }{(p-\frac{1}{2})^k(2^k-|\tau |)}\right) . \end{aligned}$$

We simplify this expression by noting $|2^k-|\tau ||= 2^k-|\tau |$, since $|\tau |\le 2^k-1$. Similarly, as $|(p-\frac{1}{2})|^k$ is symmetric around p=1/2 for p∈[0,1] and all non-negative *k*, we can restrict *p* to be in $(\frac{1}{2},1]$ giving $|(p-\frac{1}{2})|^k= (p-\frac{1}{2})^k$. Note, this restricts $(p-\frac{1}{2})^k>0$ however since $f_\circ$ is a symmetric distribution around 0 (i.e. $f_\circ (\theta )=f_\circ (-\theta )$), this restriction does not affect the convolution. Therefore, we have

29 $$\begin{aligned} f_\tau (\theta )=\frac{1}{(p-\frac{1}{2})^k(2^k-|\tau |)}f_\circ \left( \frac{\theta }{(p-\frac{1}{2})^k(2^k-|\tau |)}\right) . \end{aligned}$$

Using Eq. 26 and the expression for $f_\tau$ given above, we have30If we consider the expression inside the $f_\circ (\cdot )$ in Eq. 30, we note that as τ takes integer values $\{-(2^k-1),\ldots ,(2^k-1)\}$ and due to the absolute function, we have that τ takes every value from 1 to $2^k-1$ twice with τ=0 the only non-repeated term. Therefore, the convolution in Eq. 30 can be written using only the positive terms for τ and the power convolution, $f^{*2}$, along with the lone τ=0 term. This, and noting that $2^k\left[ (2^k-1)!\right] ^2=2^k!(2^k-1)!$, allows Eq. 30 to be written as31where each $f_\circ \left( \frac{1}{\tau (p-\frac{1}{2})^k}\theta \right)$ distribution has support $[-\pi \tau (p-\frac{1}{2})^k,\pi \tau (p-\frac{1}{2})^k]$, similar to the case for k=2 in Theorem 1.

Finally, we denote $f^{\{2^k\}}_{\circ }$ as the circular distribution found by wrapping $f^{\{2^{k}\}}(\theta )$ around the unit circle, to give a distribution with support $(-\pi ,\pi ]$; that is

32 $$\begin{aligned} f^{\{2^k\}}_{\circ }(\theta )=\sum _{m=-(2^k-1)}^{2^k-1}f^{\{2^{k}\}}(\theta +2m\pi ), \end{aligned}$$

where the limits of summation are given by noting that the support for $f^{\{2^k\}}_{\circ }(\theta )$ is found by the sum of the domains of each $f_\circ$ in Eq 31. As the value of *p* lies in (1/2, 1], the support for each $f_\circ \left( \frac{1}{\tau (p-\frac{1}{2})^k}\theta \right)$ is $[-\pi \tau (p-\frac{1}{2})^k,\pi \tau (p-\frac{1}{2})^k]\subseteq [-\pi \tau (\frac{1}{2})^k,\pi \tau (\frac{1}{2})^k]$. Recalling that τ takes all integer values in $[1,2^k-1]$, and that $f_{\circ }^{*2}$ denotes the self convolution ($f_{\circ }*f_\circ$), summing across τ gives the support of  to be contained in $2\sum _{\tau =1}^{2^k-1}[-\pi \tau (\frac{1}{2})^k,\pi \tau (\frac{1}{2})^k]=[-(2^k-1)\pi ,(2^k-1)\pi ]$. Finally, the $f_\circ \left( \frac{\theta }{2^k(p-\frac{1}{2})^k}\right)$ term has support contained in $[-\pi ,\pi ]$ and hence the support of $f^{\{2^k\}}_{\circ }(\theta )$ is contained in $[-2^k\pi ,2^k\pi ]$, and hence the limits required in the summation of Eq 32 must range from $-(2^k-1)\pi$ to $(2^k-1)\pi$ as required. □

#### Accuracy

**Fig. 7:**
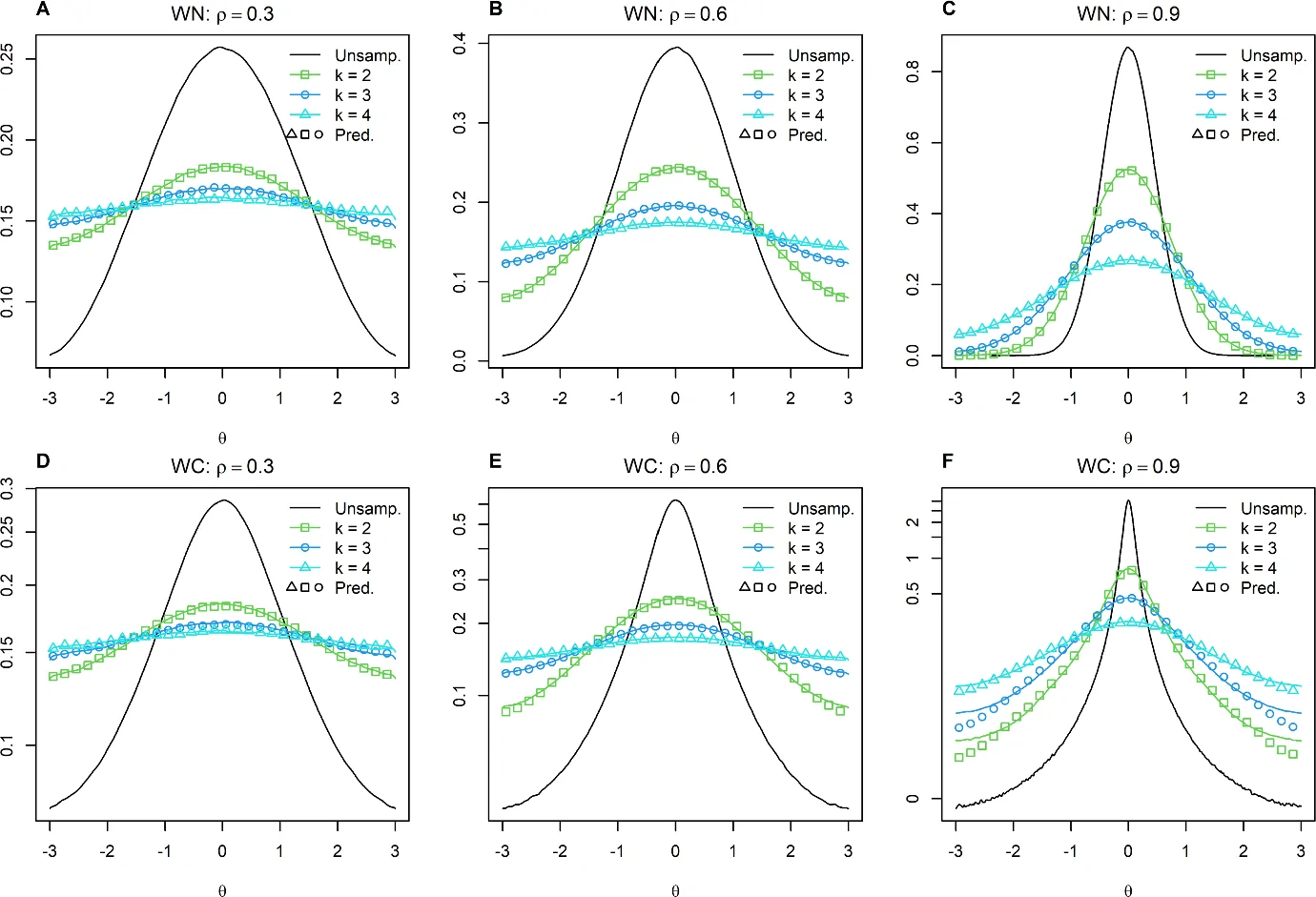
Panel showing the predicted results from Eq. 16 in Theorem 2 (points) against simulated results (solid lines). In all plots, the sub-sampling rates shown are for $r=2^k$ with k=2 (green), k=3 (blue), k=4 (cyan). The initial distributions were a wrapped normal (**A**–**C**) and a wrapped Cauchy (**D**–**F**), with mean resultant vector length of ρ=0.3 (**A**, **D**), ρ=0.6 (**B**, **E**), and ρ=0.9 (**C**, **F**). In all cases the value for *p* was estimated for each sub-sampling and ρ value. The black solid line shows the initial unsampled distribution of turning angles in the RW. Note, due to the peakedness of the wrapped Cauchy distribution, the *y*-axis has been log transformed to aid visualisation in plots (**D**–**F**). In each plot and for each sub-sampling rate, the optimal value of the parameter $p^*$ was estimated for each sub-sampling rate and ρ value by minimising the difference between the predicted curve (points) to the observed results (solid lines)—further discussion of this is given in section 3.3

Figure 7 demonstrates the accuracy of the expression for $f_{\circ }^{\{2^{k}\}}$, by comparing the approximation from Eq. 16 in Theorem 2 to simulated results across varying circular distributions and sampling rates. The largest discrepancies are apparent for angles close to $\pm \pi$ when ρ is high (see Fig. 7F). Considering the absolute percentage error (see Appendix E), the discrepancy between the simulated (solid) and theoretically predicted (point) distributions is generally very small, typically remaining within 1% to 3% across θ values and parameter combinations. These modest errors can be explained by the analytical approximation used in the theoretical derivation, and the inherent variability introduced when fitting smooth density curves to finite simulated samples. Importantly, the larger apparent percentage errors occur almost exclusively in regions where the underlying density is close to zero. In these low density tails, even small absolute differences of the order of $10^{-3}$ to $10^{-4}$, inflate the percentage error, making it appear large despite the actual discrepancy being negligible. Overall, the results demonstrate that the theoretical predictions track the simulated distributions closely, with deviations that are both quantitatively small and well understood. It should be noted that these results are sensitive to the precise value of the parameter *p* which is explored further in the following section.

Kareiva and Shigesada (1983) showed that, as the sampling rate of a correlated random walk (CRW) increases, the resulting process approaches a simple random walk, characterised by a uniform turning-angle distribution. The following corollary uses this property to provide a characterisation of the limiting behaviour for distribution $f^{\{2^k\}}(\theta )$, when $f_\circ (\theta )$ is limited to a symmetric decreasing distribution. The Corollary reduces the question to the behaviour of the product of the first trigonometric moments of the component distributions $f_\tau$, highlighting the role of the parameter *p* in determining the limiting behaviour. However, the corollary relies on assuming Conjecture 1 below.

##### Conjecture 1

Let $g_\circ (\theta )$ be a circular distribution that is symmetric about 0 and monotonically non-increasing on [0,π]. If the first trigonometric moment satisfies $\phi _1=0$, then $g_\circ =g_U$, the circular uniform distribution.

Before proceeding to the Corollary, we introduce the following Lemma regarding the distributions in Theorem 2

##### Lemma 1

If $f_\circ (\theta )$ is symmetric about 0 and monotonically non-increasing on [0,π], then each $f_\tau (\theta )$, as defined in Theorem 2, is also symmetric about 0 and monotonically non-increasing on [0,π]. Moreover, $f^{\{2^k\}}(\theta )$ is symmetric about 0 and monotonically non-increasing on [0,π].

##### Proof

From Eq. 28, as $f_\tau$ is a positive linear rescaling, of $f_\circ$, then $f_\tau$ is also symmetric about 0 and monotonically non-increasing on [0,π]. Moreover, since $f^{\{2^k\}}$ is formed by repeated convolution of $f_\tau$, which are symmetric and monotonically non-increasing, then by Wintner’s theorem (Wintner 1938; Purkayastha 1998), it follows that $f^{\{2^k\}}(\theta )$ is symmetric about 0 and monotonically non-increasing on [0,π]. □

##### Corollary 2

(of Conjecture 1) Define $f_\circ (\theta )$, $f^{\{2^k\}}(\theta )$ and $f_\tau (\theta )$ as in Theorem 2, with $f_\circ (\theta )$ symmetric about 0 and non-increasing on [0,π]. Let $\phi ^{\{2^k\}}_1$ and $\phi _1^{\{\tau \}}$ denote the first trigonometric moments of $f^{\{2^k\}}(\theta )$ and $f_\tau (\theta )$, respectively. Then

$$\begin{aligned} f^{\{2^k\}}(\theta )\rightarrow f_U(\theta ) \end{aligned}$$

as $k\rightarrow \infty$ if and only if

$$\begin{aligned} \phi _1^{\{0\}}\prod _{\tau =1}^{2^k-1}\left( \phi _1^{\{\tau \}}\right) ^2\rightarrow 0. \end{aligned}$$

##### Proof

A circular density $f_{\circ }$ has first trigonometric moment

$$\begin{aligned} \phi _1=\mathbb {E}[e^{i\theta }] =\int _{-\pi }^{\pi } e^{i\theta } f_{\circ }(\theta )\,d\theta =\mathbb {E}[\cos \theta ] + i\,\mathbb {E}[\sin \theta ], \end{aligned}$$

with mean resultant length satisfying $0\le |\phi _1|\le 1$ (see Appendix A). As $f_\circ (\theta )$ is a symmetric non-increasing circular distribution then, by Conjecture 1, the case when $|\phi _1|=0$ corresponds respectively to the uniform distribution on $[-\pi ,\pi )$, and the case when $|\phi _1|=1$ returns a point mass at the mean direction (Mardia and Jupp 2009; Fisher 1995; Jammalamadaka and Sengupta 2001). For the zero-centred symmetric densities $f_{\tau }$ from Theorem 2, symmetry implies $\mathbb {E}[\sin \theta ]=0$, hence

$$\begin{aligned} \phi ^{\{\tau \}}_1=\int _{-\pi }^{\pi }\cos \theta \, f_{\tau }(\theta )\,d\theta . \end{aligned}$$

However, since $f_{\tau }(\theta )=0$ for $\theta \notin [-a_{\tau }\pi ,a_{\tau }\pi ]$,

$$\begin{aligned} \phi ^{\{\tau \}}_1=\int _{-a_{\tau }\pi }^{a_{\tau }\pi }\cos \theta \, f_{\tau }(\theta )\,d\theta . \end{aligned}$$

Substituting $f_{\tau }(\theta )=\frac{1}{a_{\tau }} f_{\circ }(\frac{\theta }{a_{\tau }})$, where $a_{\tau }=\left( p-\frac{1}{2}\right) ^k(2^k-|\tau |)$, for $0\le \tau \le 2^k-1$ (Eq 28), yields

$$\begin{aligned} \phi ^{\{\tau \}}_1=\int _{-a_{\tau }\pi }^{a_{\tau }\pi }\frac{1}{a_{\tau }}\cos \theta \, f_{\circ }\!\left( \frac{\theta }{a_{\tau }}\right) d\theta . \end{aligned}$$

Using the change of variables $u=\theta /a_{\tau }$, gives

$$\begin{aligned} \phi ^{\{\tau \}}_1=\int _{-\pi }^{\pi }\cos (a_{\tau }u)\, f_{\circ }(u)\,du. \end{aligned}$$

Applying the triangle inequality and noting that *f*(*u*) is non-negative, gives

$$\begin{aligned} |\phi ^{\{\tau \}}_1|=\left| \int _{-\pi }^{\pi } \cos (a_\tau u)f_\circ (u)\,du\right| \le \int _{-\pi }^{\pi } |\cos (a_\tau u)|f_\circ (u)\,du\le \int _{-\pi }^{\pi } f_\circ (u)\,du=1 \end{aligned}$$

with equality only for a point mass distribution. Thus $0\le |\phi ^{\{\tau \}}_1|\le 1$.

Now, recalling that the distribution $f^{\{2^k\}}(\theta )$ is formed by the compositionThis has first trigonometric moment

33 $$\begin{aligned} \phi _1^{\{2^k\}} = \phi _1^{\{0\}}\prod _{\tau =1}^{2^k-1}\left( \phi _1^{\{\tau \}}\right) ^2, \end{aligned}$$

since trigonometric moments (equivalently, Fourier coefficients) multiply under convolution (Mardia and Jupp 2009; Fisher 1995; Jammalamadaka and Sengupta 2001). From Lemma 1, $f^{\{2^k\}}(\theta )$ is symmetric and non-increasing. Now assuming Conjecture 1 to be true, $f^{\{2^k\}}(\theta )$ tends to the circular uniform distribution as $k\rightarrow \infty$, then $\phi _1^{\{2^k\}}\rightarrow 0$ and hence from Eq. 33 we have

34 $$\begin{aligned} \phi _1^{\{0\}}\prod _{\tau =1}^{2^k-1}\left( \phi _1^{\{\tau \}}\right) ^2 \rightarrow 0, \end{aligned}$$

as required.

For the reverse implication, if we know that $\phi _1^{\{0\}}\prod _{\tau =1}^{2^k-1}\left( \phi _1^{\{\tau \}}\right) ^2\rightarrow 0$, then from Eq. 33, Lemma 1 and Conjecture 1, we have $\phi _1^{\{2^k\}}\rightarrow 0$ and hence $f^{\{2^k\}}(\theta )\rightarrow f_U$. □

It should be noted that this corollary does not show directly that $f^{\{2^k\}}(\theta )$ tends to the uniform distribution as *k* increases, rather, it demonstrates that the behaviour of $f^{\{2^k\}}(\theta )$ in this scenario is governed by the behaviour of $\phi _1^{\{0\}}\prod _{\tau =1}^{2^k-1}\left( \phi _1^{\{\tau \}}\right) ^2$. Although each individual moment satisfies $|\phi _1^{\{\tau \}}|<1$ for finite *k*, the moments themselves depend on both *k* and the parameter *p* through Eq. 28. Therefore, convergence of the product is not fully determined. Understanding the precise dependence of this limiting behaviour on *p* requires a more detailed analysis of this product, as does a proof of Conjecture 1 which is beyond the scope of the present work.

### The parameter *p*

Here we briefly explore the tuning parameter *p* used within Eq. 16 from Theorem 2. As discussed, the parameter *p* is linked to the geometry of the underlying RW, which for a general CRW can be described by the ‘sinuosity’ of the movement (Benhamou 2004). Therefore, the parameter *p* must encode information about the initial step-length and turning angle distributions as well as accounting for auto-correlation inherited in the process from the sub-sampling (Nams 2013).

This can be seen by considering sub-sampling at rate r=2 in Theorem 2 and comparing this to Theorem 1. Setting r=2 and therefore k=1 in Eq. 16 from Theorem 2, should return Eq. 5 from Theorem 1. Doing so gives

$$\begin{aligned} f^{\{2\}}(\theta )=\frac{1}{2(p-\frac{1}{2})^3}\left[ f_\circ \left( \frac{\theta }{2(p-\frac{1}{2})}\right) *f_{\circ }^{*2}\left( \frac{\theta }{(p-\frac{1}{2})}\right) \right] . \end{aligned}$$

Letting p=1, gives

$$\begin{aligned} f^{\{2\}}(\theta )=4\left[ f_\circ \left( \theta \right) *f_{\circ }^{*2}\left( 2\theta \right) \right] . \end{aligned}$$

as required.

This demonstrates that when the sampling rate is r=2 and the initial underlying step-length distribution is constant, as in Theorem 1, we have p=1. For differing initial step-length distributions the precise value of *p* may vary slightly. Whilst Appendix C demonstrates that the precise step-length distribution does not greatly influence the given turning angle distribution after sampling, the parameter *p* would encode any slight effect that does occur.

For each combination of wrapped distribution and concentration parameter, a correlated random walk of order $10^6$ steps was simulated and sub-sampled at rates r=4,8,16. The resulting empirical turning angle densities were smoothed using ‘approxfun’ in ‘*R*’, and these were compared with the theoretical functions $f^{\{2^k\}}$ from Theorem 2 by varying *p* over a fine grid (step size 0.001). Following the approach of Bailey and Codling (2021), the optimal value, $p^*$ was defined as the value of *p* that minimised the mean squared $L^2$ distance between the empirical and theoretical densities across θ, evaluated from -π to π at 0.001 intervals. The final estimate of $p^*$ for each (ρ,r) combination was taken as the average across 100 repeatedsimulations.

**Fig. 8:**
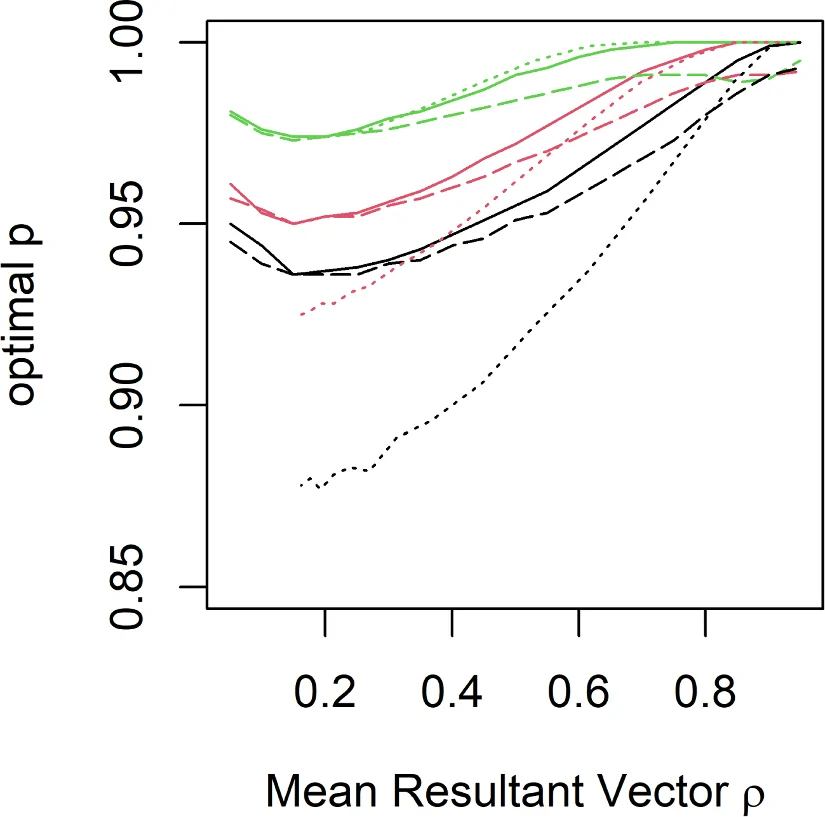
Optimal *p* values as a function of the length of the mean resultant vector ρ. Colours denote different sub-sampling levels: k=2—green; k=3—red; k=4—black. Line type denotes different distributions: solid—wrapped normal; dashed—wrapped Cauchy; dotted—wrapped triangle

**Fig. 9:**
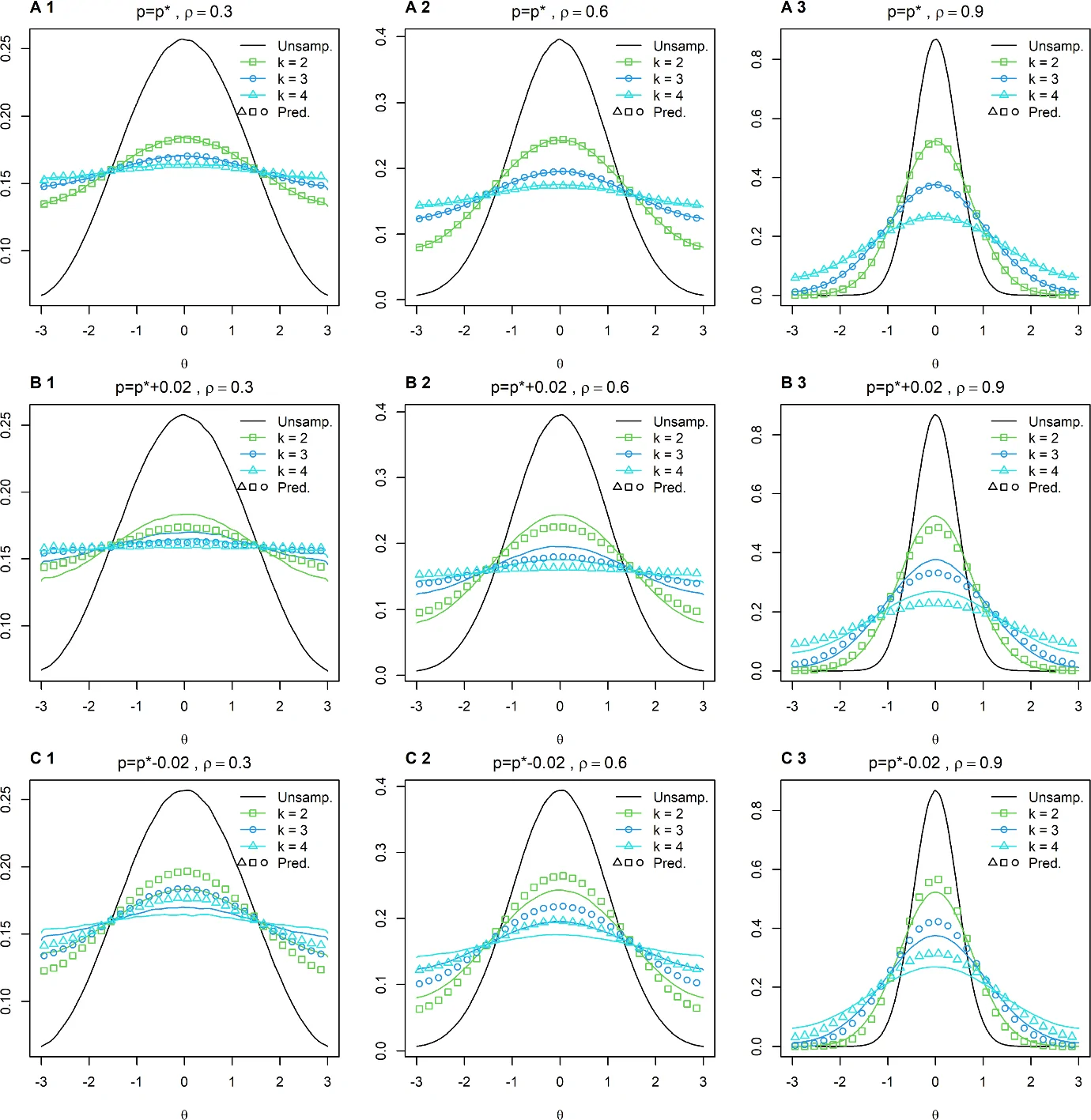
Panel demonstrating the sensitivity of the predicted distributions as given in Theorem 2 to changes in the value of the parameter *p*. The value of *p* took the optimal value $p^*$ as calculated in Fig. 8A1–A3; the optimal value plus 0.02 (**B1**–**B3**); and the optimal value minus 0.02 (C1-C3). Results are for the initial CRW featuring turning angles described by a wrapped Normal distribution (black line) and fixed step-length distribution. Colours correspond to differing levels of sub-sampling: green - r=2; cyan - r=4; dark blue—r=8. In all cases, observed results are in solid lines, results given by Theorem 2 are points

Results are shown in Fig. 8, and demonstrate that the value of *p* is sensitive to the sampling rate (coloured lines) and to the underlying turning angle distribution (type of line). It is noticeable that the wrapped normal and wrapped Cauchy give similar result, compared to the wrapped triangle. Given that both the wrapped normal and wrapped Cauchy are two of the most utilised and observed distributions in movement data (both coming from the family of symmetric wrapped stable distributions (Jammalamadaka and Sengupta 2001); see Appendix A), this indicates that it is the sampling rate and concentration parameter that are the most important factors to consider when determining the optimal *p* value. Whilst it is also apparent that the value of *p* mostly lies in (0.94, 1), the sensitivity of Theorem 2 to the precise value of *p* is demonstrated in Fig. 9, which shows the sensitivity of the distributions to the parameter *p* by fixing the value of *p* and comparing the predicted (points) with the observed results (solid lines). This shows that a relatively small perturbation away from the optimal value for *p* of order ±0.02 can have large effects on the accuracy.

## Applications

Due to their ease of use, their interpretability and natural fit to telemetry data, discrete-time continuous space random walks have been shown to be an important tool in the analysis of animal movement and in the field of movement ecology (Kareiva and Shigesada 1983; Nathan et al. 2008; Codling et al. 2008; Bailey et al. 2018; Codling et al. 2024), driving the development of new theory (Codling et al. 2008; Smouse et al. 2010; Lutscher and Hillen 2021) and deepening our understanding of complex ecological processes (Berg 1993; Hooten et al. 2017; Lewis et al. 2013).

Here we present two examples of how comparing the expected sub-sampled turning angle distributions to that found from observed data, can be used to qualitatively analyse movement data.

### Determine between the presence of a CRW and a BRW

First, we consider determining between trajectories which are either formed by a biased random walk (BRW) or CRW. Recall, a CRW can be defined by the step-length and turning angle, θ, whereas in comparison, a BRW where the bias is assumed to be towards a constant direction (point at infinity), can be described by the step-length and *orientation*, ϕ, which is the angle taken relative to some absolute direction; usually either a bearing from North, or from the positive *x*-axis (Codling et al. 2008; Bailey et al. 2018). In this case we would have ϕ being described by a circular distribution, $g_{\circ }$, parameterised by, μ, the direction of the bias in relation to the absolute direction of measure and $\rho _{\text {BRW}}$, the length of the mean resultant vector in the direction of μ. Similar to a CRW, the MRV, $\rho _\text {BRW}$ determines the ‘strength’ of the bias with values closer to 1 giving more straight-line movement, and values closer to 0 giving more meandering movement. Despite not being characterised by the turning angles, a BRW formulated in this way still maintains a turning angle distribution described by some zero centred circular distribution. Therefore identifying whether movement is best described by a BRW or a CRW cannot simply be determined by considering the turning angle distribution.

There are various methods to determine whether recorded movement is best described by a BRW or a CRW (Benhamou 2006; Marsh and Jones 1988). However, these methods are not always reliable nor practical, often resulting in large variances in the measured statistic, the need to compare the measured statistic from observed data with that of large scale simulations, or an over reliance on best fitting distributions to data sets, as well as requiring a trajectory featuring a large number of steps Benhamou (2006). Here we consider short paths and demonstrate that by simply comparing the observed distribution of turning angles when a movement path is sub-sampled, with the expected distribution as predicted by Theorem 2, gives a simple qualitative analysis of whether a CRW or BRW is the best descriptive model.

**Fig. 10:**
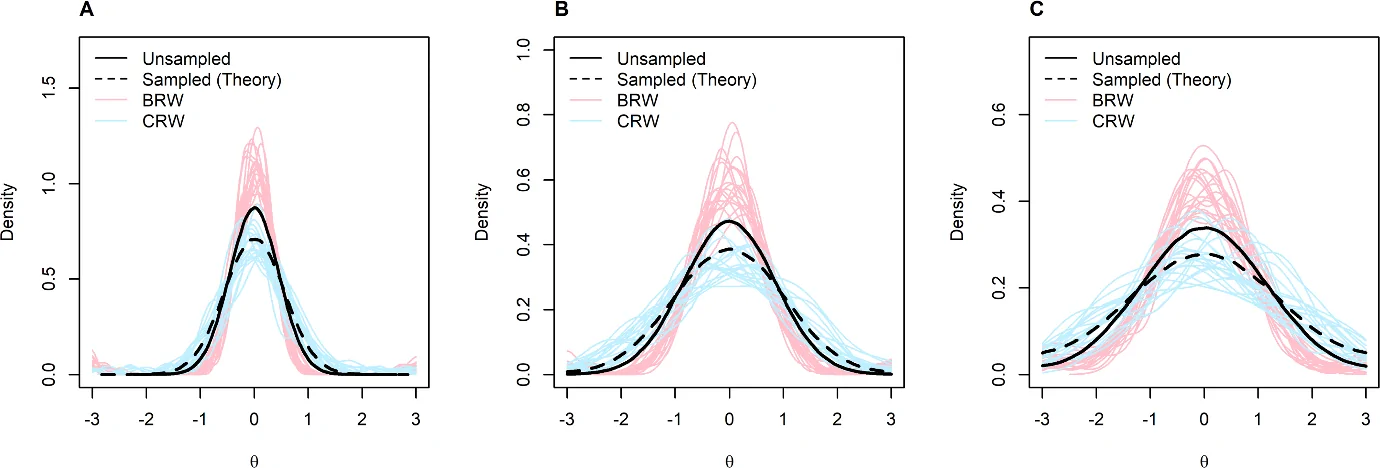
Comparison of sub-sampled turning-angle distributions for short trajectories generated using either a BRW or CRW model. Trajectories of 100 steps were generated with unit step lengths. For CRWs, turning angles were drawn from zero-centred wrapped normal distributions with $\rho _\text {CRW}=0.9,0.7,0.5$ (Panels **A**, **B**, **C** respectively). For BRWs, orientations ϕ were drawn from zero-centred wrapped normal distributions with $\rho _\text {BRW}=0.95,0.84,0.71$ (Panels **A**, **B**, **C** respectively). Each trajectory was sub-sampled at rate r=2. Each panel shows the fitted turning angle distributions for: unsampled trajectory (solid black); expected sub-sampled CRW distribution from Theorem 1 (black dashed); sub-sampled BRW (pale red); sub-sampled CRW (pale blue). The procedure was repeated 20 times for each movement type

#### Simulated data

Trajectories of length 100 steps were simulated, formed either using a BRW or CRW model. In each case, step-lengths were fixed at unit length, with the turning angles described by a zero-centred wrapped normal distribution with $\rho _{\text {CRW}}=0.9, 0.7, 0.5$ for the CRW, and orientations, ϕ described by a zero-centred wrapped normal distribution with $\rho _{\text {BRW}}=0.95, 0.84, 0.71$. These gave the BRW a turning angle distribution equivalent to the values chosen for $\rho _{\text {CRW}}$, therefore, each trajectory, whether BRW or CRW, was expected to have the same step length and turning angle distributions.

Figure 10 demonstrates that if the sub-sampled distribution of turning angles has a sharper peak than the original distribution, the movement is best described by a BRW, whereas a CRW is expected to have a flatter peak, below the un-sampled turning angle distribution. The clear delineation between the results from the BRW (red) and the CRW (blue) indicate that this method can be used on short movement paths to provide an indication of whether movement is likely from a BRW or CRW.

Although these results do not provide a quantitative measure for distinguishing BRWs from CRWs, they show a clear qualitative diagnostic that does not depend on extensive simulation or formal model fitting. However, letting $\rho _\text {unsamp}$ denote the mean resultant vector of the original trajectory and $\rho _\text {samp}$ the mean resultant vector of the sub-sampled trajectory, a simple quantitative rule-of-thumb states that trajectories with $\rho _\text {samp}>\rho _\text {unsamp}$ indicate BRW-like behaviour, whereas $\rho _\text {unsamp}>\rho _\text {samp}$ suggests CRW-like movement. Developing this observation into a formal statistical test would require additional work and lies beyond the scope of the present study.

#### Observed data—*P. cupreus*

**Fig. 11:**
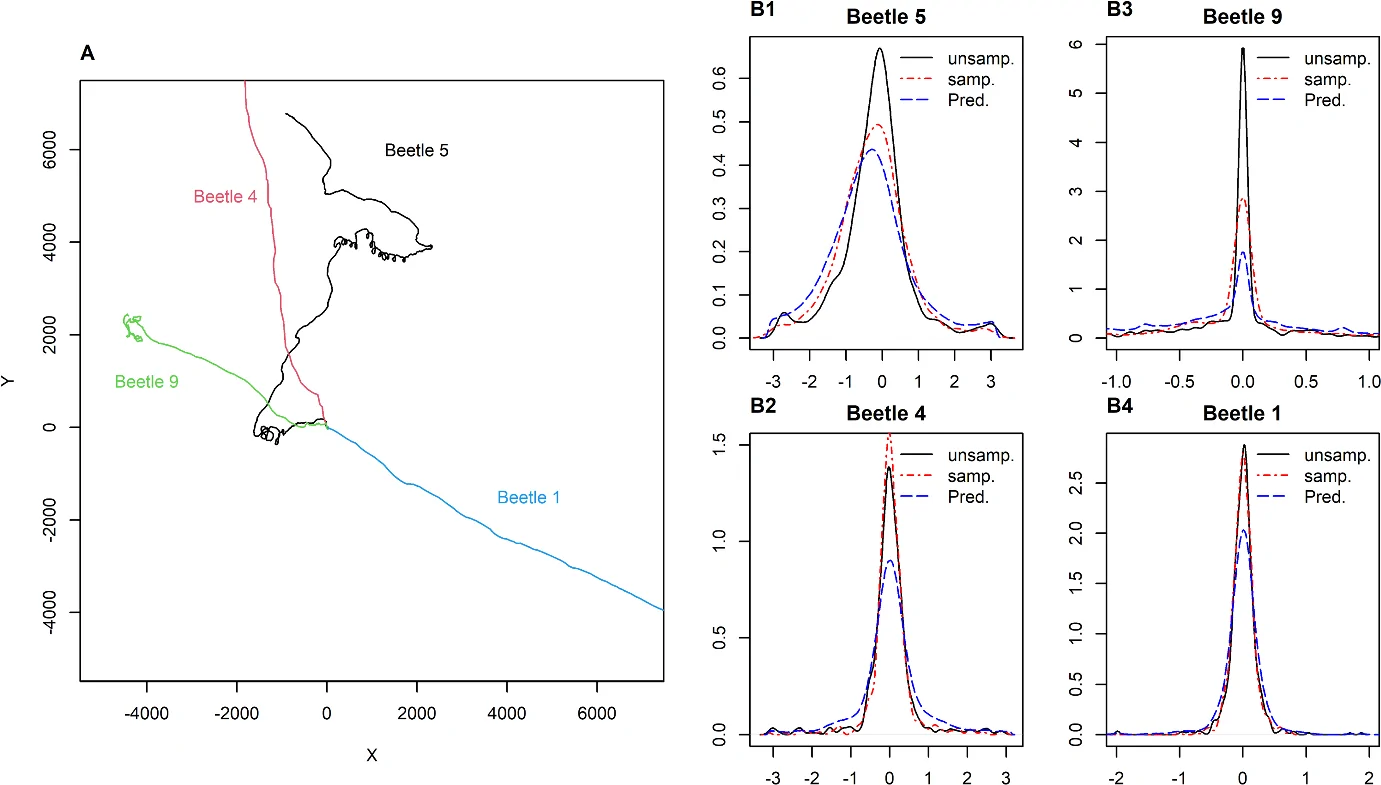
**A** depicts the movement trajectories of the four *P. cupreus* beetles as described in Section 3.1.2 and by Bailey et al. (2021a). **B1-4** shows the turning angle distribution of the un-sampled paths (black, solid line), the turning angle distribution found when sub-sampling the trajectories as a rate of r=2 (red, dashed-dotted line), against the expected sub-sampled turning angle distribution as calculated using Eq. 9 (blue, dashed line)

We now demonstrate this approach on observed experimental movement data. In Bailey et al. (2021a) *P. cupreus* beetles were tracked with their resulting movement classified as either a BRW, CRW or undetermined using the Marsh-Jones statistic (Marsh and Jones 1988). That approach requires multiple repeated simulation to produce a statistic to give a quantitative indication as to which RW model best describes the data, however, the resulting statistic can produce wide confidence intervals, which was compounded in this instance by the relative small data set, with each trajectory consisting of 300 time steps. We can compare the results found in Bailey et al. (2021a) using the Marsh-Jones statistic with the method discussed above. Figure 11 shows the recorded movement paths (A) for four beetles, along with the distribution of the turning angles (B—solid black line). In the original manuscript, the movement from Beetle 5 and Beetle 9 were classified as a CRW, Beetle 4 a BRW and Beetle 1 was undetermined (see Supplementary Material File 1 in Bailey et al. (2021a)). If we consider the sub-sampled turning angle distributions (dashed-dotted red line) and compare these with the expected result for a pure CRW (dashed blue line), we can see that Beetle 5 and Beetle 9 seem closely related to the predicted results indicating a CRW is potentially an appropriate model. Whereas, Beetle 4 has a much sharper peak than the initial distribution and therefore would be best described by a BRW. Beetle 1 has a sub-sampled distribution very similar to the initial distribution and therefore can not be reliable described by either a BRW or CRW. These results therefore match those found in the initial analysis, but using a simpler approach.

### Identification of multi-state or more complex movement

Recorded movement often covers multiple behaviours in individuals. For example, animals can exhibit distinct movement phases across a period of time such as foraging, which can be characterised by slow winding movement, and travelling, where movement is faster and more directed (Gurarie et al. 2016). These can be identified using various methods, including Hidden Markov models (Michelot et al. 2016), break point analyses (Edelhoff et al. 2016) and mixed distributions (Bailey and Codling 2021). However, as discussed in Bailey and Codling (2021) it is not always clear when such a multi-state model should be considered by examining the data. Here we show how by considering the sub-sampled turning angle distribution of observed movement data, an indication that a multi-state model would be best suited to the data rather than a simple single-state RW model, can be inferred. This approach does not give specific information regarding the multi-state model, such as the number of states, the shape of the distributions in each state or the time spent in each state; rather it can be used as a first pass to help indicate whether a more in-depth analysis to determine the best fitting multi-state model should be used. With the approach requiring a relatively small number of data points to give a clear indication.

**Fig. 12:**
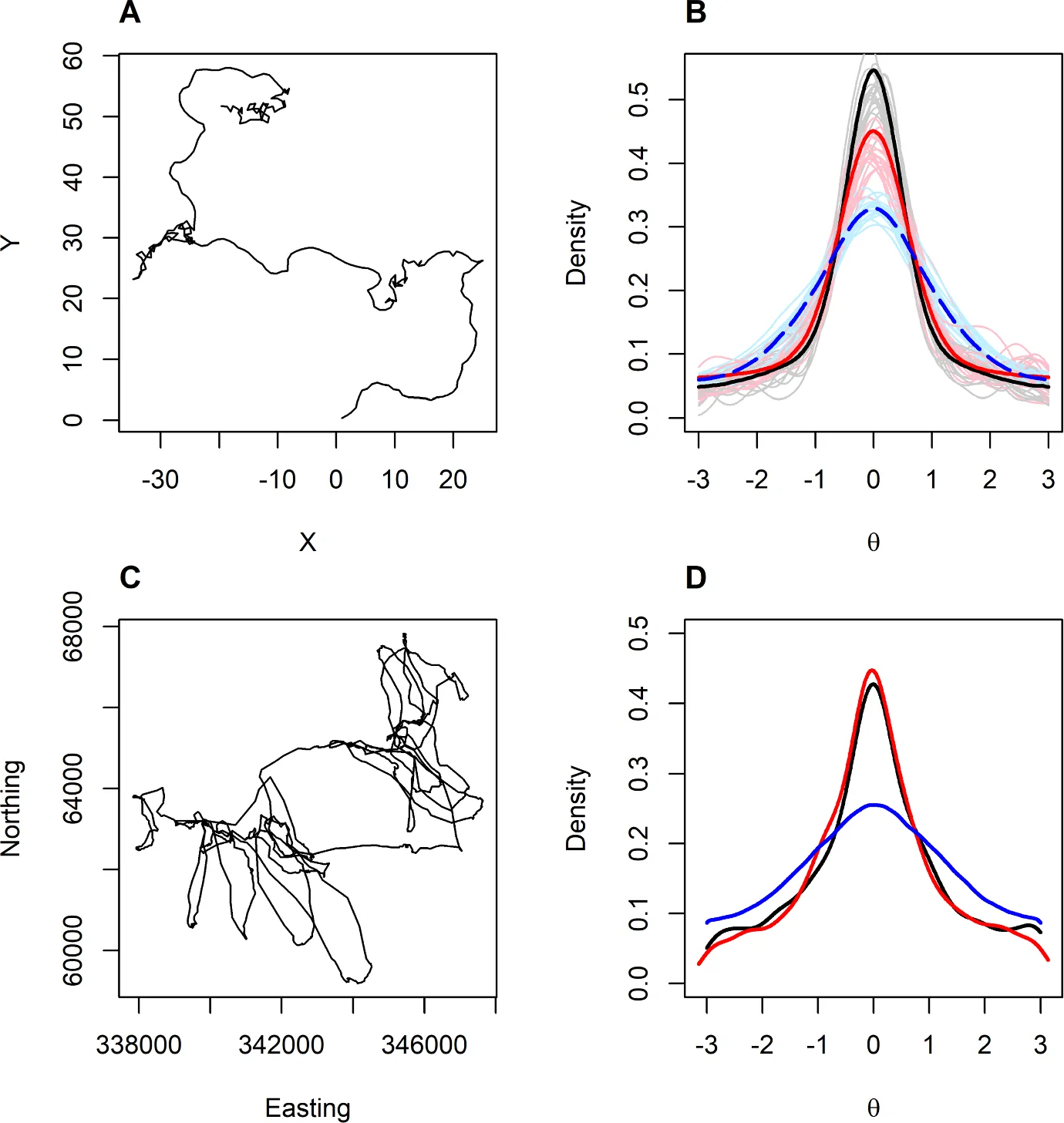
**(A)** Depicts an example of a multi-state movement path featuring two distinct movement phases. **(B)** Plots the turning angle distributions for the multi-state movement depicted in (**A**); the distribution from the unsampled movement path (black), the distribution from the sub-sampled movement (red) and the expected distribution for the sub-sampled path assuming movement was formed from a single CRW (blue). The lighter lines correspond to individual simulations, the bold lines correspond the the expected results. **(C)** Plots the movement path of the African elephant (ID: Haliba) from Wall et al. (2014). **(D)** plots the distribution of the turning angles from **(C)** from the un-sampled movement (black), the sub-sampled movement (red) sampled at rate r=2, and the expected sub-sampled distribution if the initial movement was from a single CRW (blue)

#### Simulated data

Figure 12A shows an example of a movement path with two distinct movement states: state 1, characterised by straighter movement (corresponding to a turning angle distribution with a high mean resultant vector, ρ); and state 2, featuring more winding movement (corresponding to a turning angle distribution with a low mean resultant vector). The corresponding turning angle distribution for the entire movement path is shown in Fig. 12B (black lines). Using this distribution, the expected distribution for turning angles from the sub-sampled path can be found using Eq. 9 (dashed blue line), this can then be compared to the distribution of turning angles from the sub-sampled movement path (red lines). Figure 12B shows the results from repeatedly simulating a mixed movement path of total length 300 steps, formed by repeated periods of 50 steps in state 1 (straighter movement) followed by 50 steps in state 2 (winding movement). The lighter lines in Figure 12B show the results from individual simulations, with the thick lines showing the averaged results from the repeated simulations. These results indicate that when movement is formed from a multi-state CRW the sub-sampled turning angle distribution (red solid) features a peak lying below the original un-sampled turning angle distribution (black solid) but above the expected sub-sampled distribution for a pure CRW (blue dashed).

#### Observed data—African Elephant

As an example with real-world data we consider the movement path of a bull African elephant, *Loxodonta africana*, (Fig. 12C) which was demonstrated by Bailey and Codling (2021) to be best described by a multi-state CRW (data available from Wall et al. (2014)). However, as discussed by Bailey and Codling (2021), the turning angle distribution of the entire movement path is well described by a single distribution and could therefore be misclassified as a single movement state. Whereas Bailey and Codling (2021) demonstrated the better fitting multi-state CRW model by performing a parameter sweep requiring large scale simulations, here we indicate the presence of such multi-sate behaviour by sub-sampling. Figure 12D shows the distribution of the turning angles from the observed data (black) along with the distribution of the sub-sampled turning angles (red) and the expected distribution of the sub-sampled turning angles when using Theorem 2 (blue). The results indicate that the movement is not well described by a pure CRW as the blue and red lines are not closely matched, however as the initial and sub-sampled distributions are closely matched (red and black) this would indicate that a more complex movement model should be explored.

## Discussion

Here we have investigated the effect of sub-sampling on the descriptive probability distributions of correlated random walks. We have given precise formulations for the step-length and turning angle distributions when a CRW is sub-sampled at a rate of every other location (r=2) assuming the initial step sizes were all constant. We have further shown it is possible to determine (up to a very close approximation) the form of the PDF for the turning angles of a sub-sampled CRW for any sub-sampling rate of the form $r=2^k$. We have demonstrated the usefulness of sub-sampling as a form of data analysis by highlighting approaches where it can help in identifying the most appropriate model to describe movement in several scenarios, with our findings having applications in various fields across the sciences, specifically in the field of movement ecology.

There are many ways this work could be advanced such as by obtaining expressions for sampling at any rate *r*. Our presented method works only for powers of 2, and cannot be extended to allow for all integer sampling rates, therefore some alternative method that does not rely on triangulation may be required. We also did not consider extending this process to CRWs in 3-dimensions. This is an active area of research, due to the increasing accuracy and availability of telemetry data recorded in 3-dimensions, this has lead to many recent advances in developing the mathematical properties of 3-dimensional CRW (Ahmed et al. 2023; Sadjadi et al. 2015).

Similarly, there is more work required to include the precise effect of differing step-length distribution. Whilst here the effect of differing initial step-lengths were not considered, as they were found to have a negligible effect on the result compared to the sample rate, turning angle distribution and mean-resultant vector of the turning angle distribution (see Appendix C), it is clear that there is an effect. More importantly, there will be auto-correlation between step-lengths and turning angles that are not considered here at higher sampling rates, rather, all sub-sampled paths were still assumed to consist of turning angles and step-lengths that are i.i.d as well as neither auto- nor cross-correlated. This has been explored when considering uniform turning angle distributions in Rosser et al. (2013), but including more general turning angle distributions and sampling at any level is yet to be solved.

Another natural extension would be to consider the effects of sampling on other, more complex, RW models, as briefly explored in the Applications (Sect. 4). When considering animal movement, individuals will often have both a preferred overall direction of movement (bias) and a preference in following their previous direction (persistence), this gives rise to the biased and correlated random walk (BCRW) and is usually characterised by some balancing combination of a BRW and a CRW. These can be created in a variety of approaches, such as vector weighted (Benhamou 1992; Bailey et al. 2018), weighted angular (Schultz and Crone 2001) or through a switching process (Peleg and Mahadevan 2016). Similarly, there are many other approaches used to describe motion in general, from across the sciences, in both discrete and continuous time, such as velocity-jump (Monmarché et al. 2020; Othmer and Hillen 2000), Ornstein-Uhlenbeck (Sevilla et al. 2019; Breed et al. 2017), continuous-time correlated random walks (Johnson et al. 2008), (truncated) Lévy walks (Humphries et al. 2013; Zaburdaev et al. 2015), persistent turner walks (Gautrais et al. 2009), along with more statistical approaches such as step-selection functions (Thurfjell et al. 2014; Duchesne et al. 2015), Brownian bridges (Horne et al. 2007) and many others. All these have different formulations which inform and describe the subsequent movement path, though the connections between models are being explored (Duchesne et al. 2015). Therefore the effect sampling has on these underlying parameters and models has yet to be fully explored outside of the discrete time RW model. Specifically, the effect of transforming a continuous movement path, to discrete paths is known to have significant effects on the analysis of the movement, therefore understanding more precisely how the frequency of this discretisation and the links between different discretisations would help retain the important information of the movement path (Mcclintock et al. 2014).

We also only considered that the initial CRW was described by one turning angle and one step-length distribution, whereas as discussed in the Applications (Sect. 4), this is usually not the case in movement data, with movement being formed from the combination of multiple movement models, each with their own parametrisation.

Animal movement near boundaries, whether physical barriers or home range edges, is well known to differ from movement within the domain, and this has important implications for how movement should be modelled. Empirical studies show that individuals often alter step lengths, turning behaviour, or dispersal decisions when approaching habitat edges or home range limits (Schtickzelle and Baguette 2003; Reeve et al. 2008), and models likewise demonstrate that boundary specific movement rules can generate markedly different spatial patterns (Jeanson et al. 2003; Tischendorf and Wissel 1997). The way boundaries are implemented in movement models, whether through reflecting, absorbing, or more complex behavioural boundary conditions, can substantially influence inferred parameters and predicted trajectories, as highlighted by reflected SDE approaches (Hanks et al. 2017) and recent work showing that boundary interactions can alter observed step length distributions (Cocconi et al. 2021). These issues are important to consider, and future work should examine them more explicitly, including how sub-sampling may bias observations of individuals near boundaries, however, such an investigation lies beyond the scope of the present study.

Whilst we showed this method of considering the sub-sampled distributions can give an indication as to whether multiple states of movement are present, as well as whether movement is best described by a CRW or a BRW this is only a qualitative analysis. Further work could look at using this methodology to provide a metric or quantitative measure as to the likely model between telemetry data, perhaps similar to the Marsh-Jones statistic (Marsh and Jones 1988).

Whilst we have extended the understanding of the effects of sub-sampling, our results in Theorems 1 & 2 lead to the following conjectures about the precise connection between the distributions of turning angles from sub-sampled CRW trajectories.

### Conjecture 2

There is no circular distribution $f_\circ (\theta ;\lambda _1,\ldots ,\lambda _n)$, other than the circular uniform distribution, such that $f^{\{2\}}_{\circ }=f_\circ (\theta ;\hat{\lambda }_1,\ldots ,\hat{\lambda }_n)$, regardless of step-length distribution Λ.

### Conjecture 3

There is no circular distribution $f_\circ (\theta ;\lambda _1,\ldots ,\lambda _n)$, other than the circular uniform distribution, such that $f^{\{r\}}_{\circ }=f_\circ (\theta ;\hat{\lambda }_1,\ldots ,\hat{\lambda }_n)$, where r≥2 an integer, regardless of step-length distribution Λ.

Conjecture 3 is not one that this work can necessarily answer as we only provide an expression for $f_\circ ^{\{r\}}$ up to close approximation. However, Conjecture 2 may follow as a direct consequence of Theorem 1 and it is therefore likely that similarly no higher sampling rate would work.

**Fig. 13:**
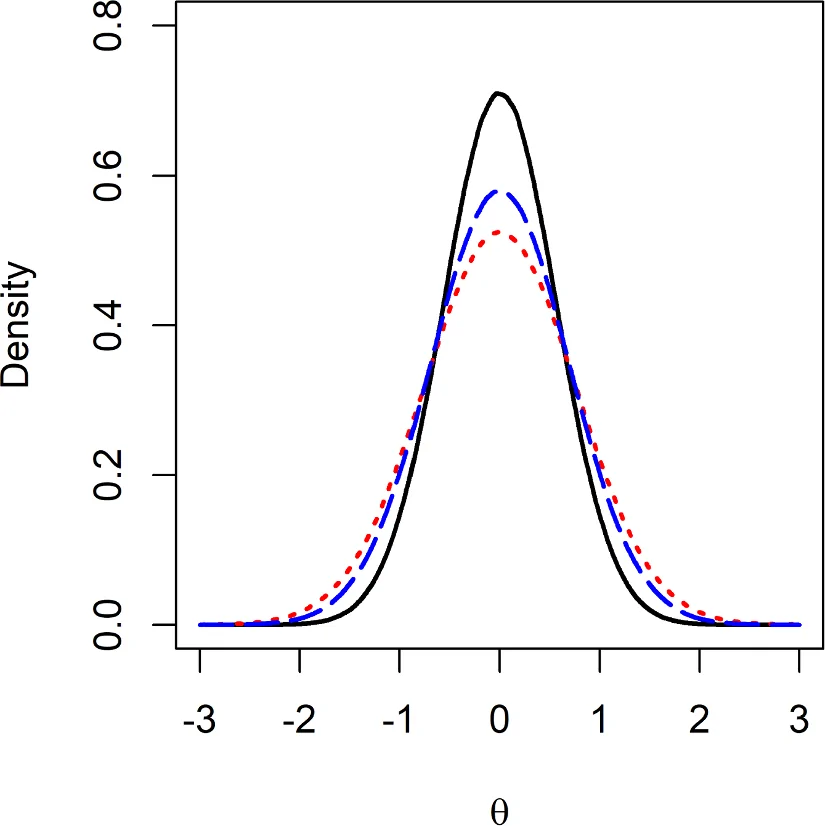
Plot showing the distribution of turning angles when a CRW is sampled at a rate of r=2 (black—solid), along with the distribution of turning angles when the same CRW is sampled at rate r=4 (red—dotted), which is compared with the distribution found when a CRW with turning angle distribution given by the solid black line is sampled at a rate of r=2 (blue—dashed), along with the corresponding step-length distribution given by the CRW corresponding to the solid black distribution

One interesting outcome from our work is that it demonstrates the following. Let $(X)_i$ be a CRW with turning angle distribution $f_{\circ ,X}$ and step-length distribution $\Lambda _X$, then from Theorem 1 we know that the subsequent RW formed from sampling $(X)_i$ at a rate of r=2 has turning angle distribution $f^{\{2\}}_{\circ ,X}$ and step-length distribution $\Lambda ^{\{2\}}_X$. Now let $(Y)_j$ be a CRW with turning angle distribution $f_{\circ ,Y}=f^{\{2\}}_{\circ ,X}$ and step-length distribution $\Lambda _Y=\Lambda ^{\{2\}}$, then the RW found by sampling $(Y)_j$ at a rate of r=2 will not yield the same distribution as that given by sub-sampling $(X)_i$ at a rate of r=4. That is $f^{\{2\}}_{\circ ,Y}\ne f^{\{4\}}_{\circ ,X}$ (see Fig. 13). In theory this could be used to determine if a CRW has previously been sampled, although in practice this will have limited uses as data rarely is known to come from a precise CRW process. However, this could highlight the importance of auto-correlation in the sampled RW, as demonstrated by Nams (2013), the act of sampling a purely CRW imparts auto-correlation between the step-lengths and turning angles into the resulting sampled RW, therefore this could be what drives this difference. If so, our method demonstrates the mathematical reasons that underpin this observation giving a quantitative measure of the difference the added auto-correlation causes to the turning angle and step-length distributions. More work could be done to understand the precise mathematical description between sampling and auto-correlation.

Overall, the CRW remains a useful and powerful tool for modelling a variety of natural processes and despite its ubiquity across the sciences there are still many fundamental mathematical and statistical properties that we do not fully understand, including those pertaining to the effects and usefulness of sub-sampling. Here we have looked to address some of these properties, however, as discussed our work leads to more questions and points to the need for continued research into CRW and RW theory in general, to help us more fully understand, model and predict these natural processes and phenomena.

## Figures and Tables

**Fig. 14:**
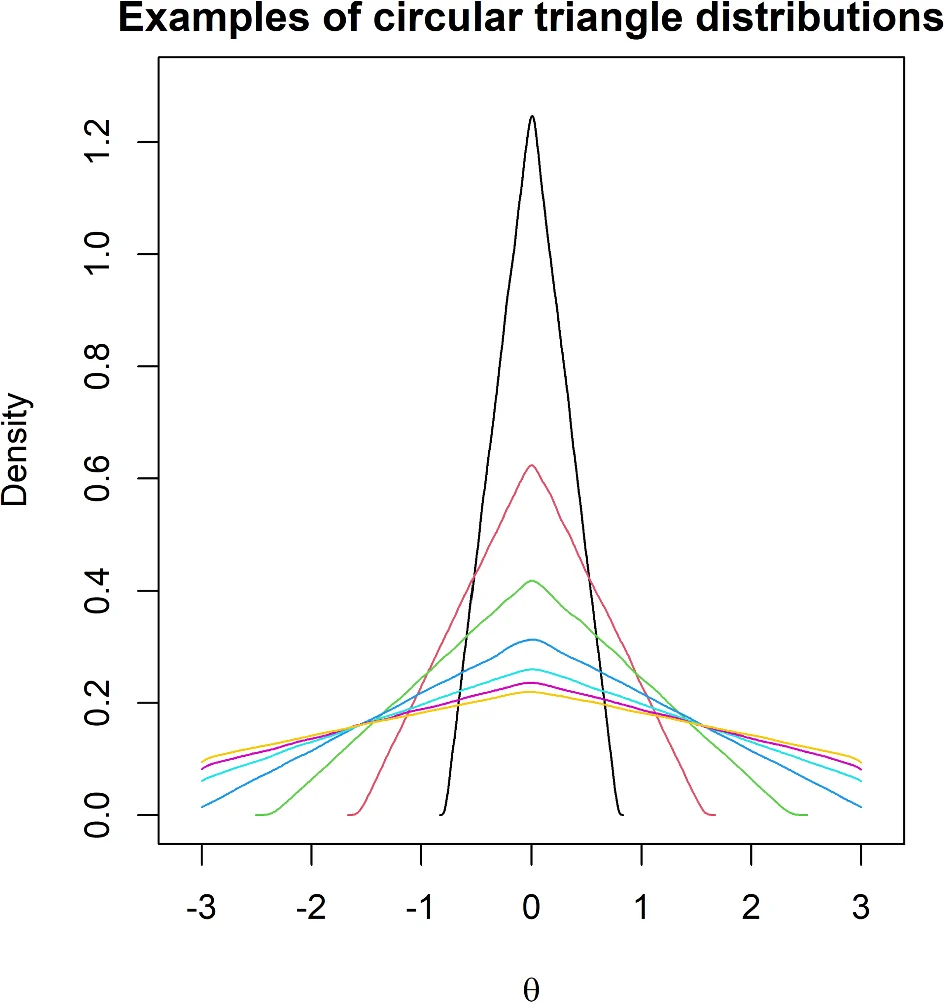
Figure showing examples of the triangle distribution used throughout the text. Lines correspond to different values of *a* which is discussed in the text in Appendix E. Here we have: $a=\frac{\pi }{4}$—black; $a=\frac{2\pi }{4}$—red; $a=\frac{3\pi }{4}$—green; a=π—blue; $a=\frac{5\pi }{4}$—cyan; $a=\frac{6\pi }{4}$—pink; $a=\frac{7\pi }{4}$—gold

**Fig. 15:**
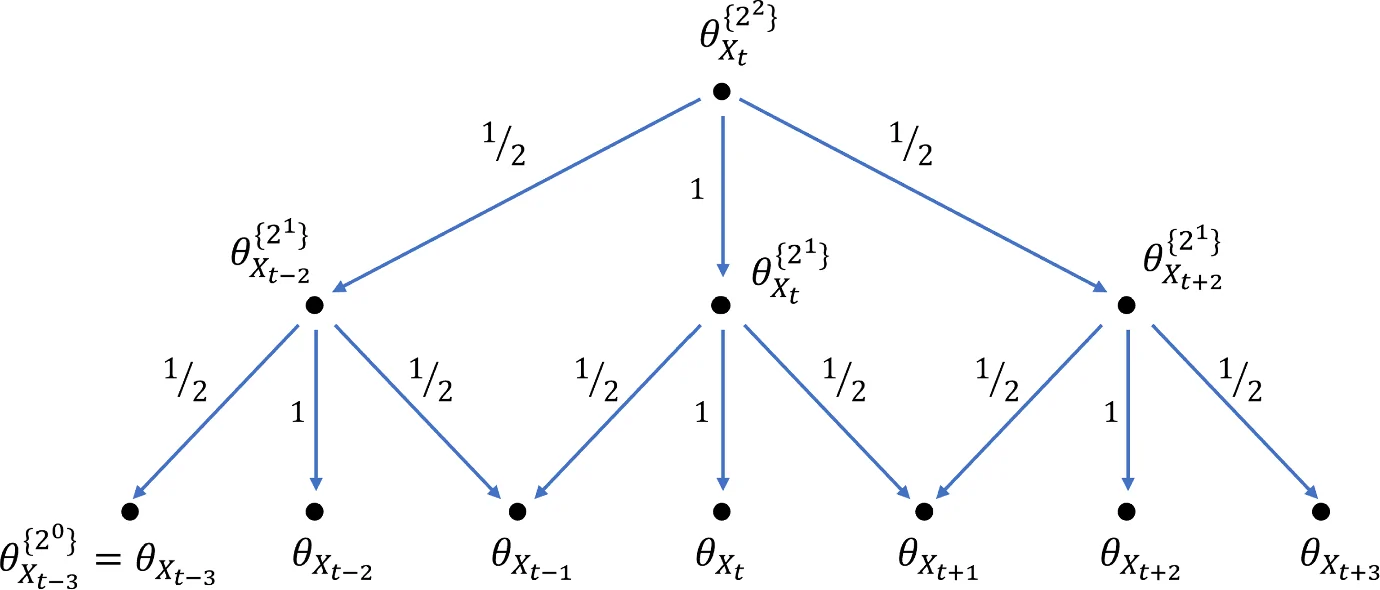
Graphical representation of the calculation for $\theta ^{\{2^2\}}_{X_t}$, similar to Fig. 6 from the Main text. The diagram demonstrates two iterations of Eq. 8 and shows that $\theta ^{\{2^2\}}_{X_t}$ can be written as the linear sum of terms $a^{\{2^2\}}_{t-\tau }\theta _{X_{t-\tau }}$ with $\tau =\{-3,-2,\ldots ,3\}$, where the coefficients $a^{\{2^2\}}_{t-\tau }$ are determined by considering every path from the top node of $\theta ^{\{2^2\}}_{X_t}$ to the leaf node $\theta _{X_{t-\tau }}$ (Eq. A.8)

**Fig. 16:**
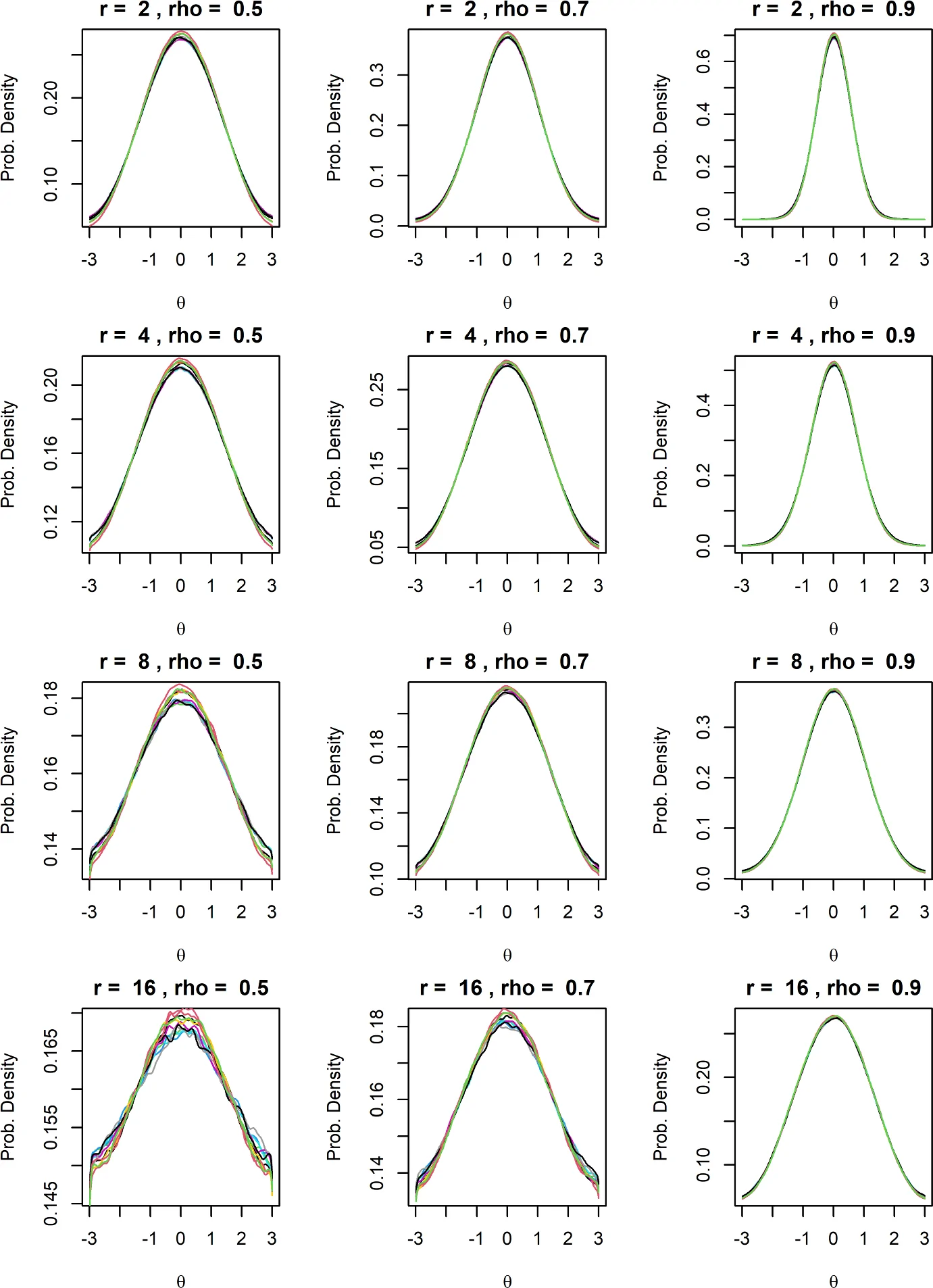
Figure showing the resulting distribution of turning angles when a RW is sampled at rate r=2,4,8,16 (rows), with initial turning angle distribution given by a zero-centred wrapped normal distribution with mean resultant vector, ρ, taking values of ρ=0.5,0.7,0.9 (columns). In all cases lines correspond to different initial step length distributions as detailed in Table 2

**Fig. 17:**
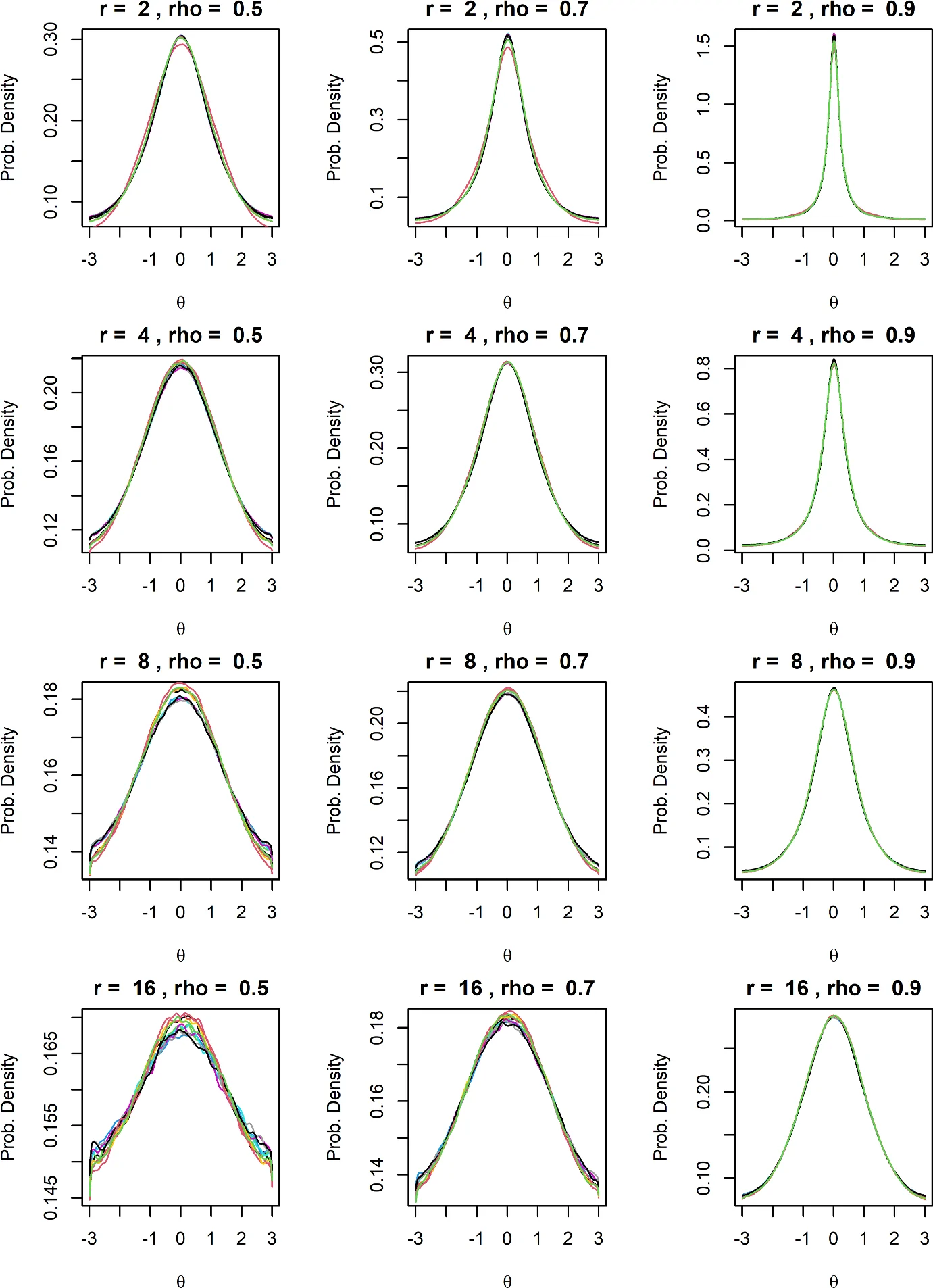
Figure showing the resulting distribution of turning angles when a RW is sampled at rate r=2,4,8,16 (rows), with initial turning angle distribution given by a zero-centred wrapped Cauchy distribution with mean resultant vector, ρ, taking values of ρ=0.5,0.7,0.9 (columns). In all cases lines correspond to different initial step length distributions as detailed in 2

**Fig. 18:**
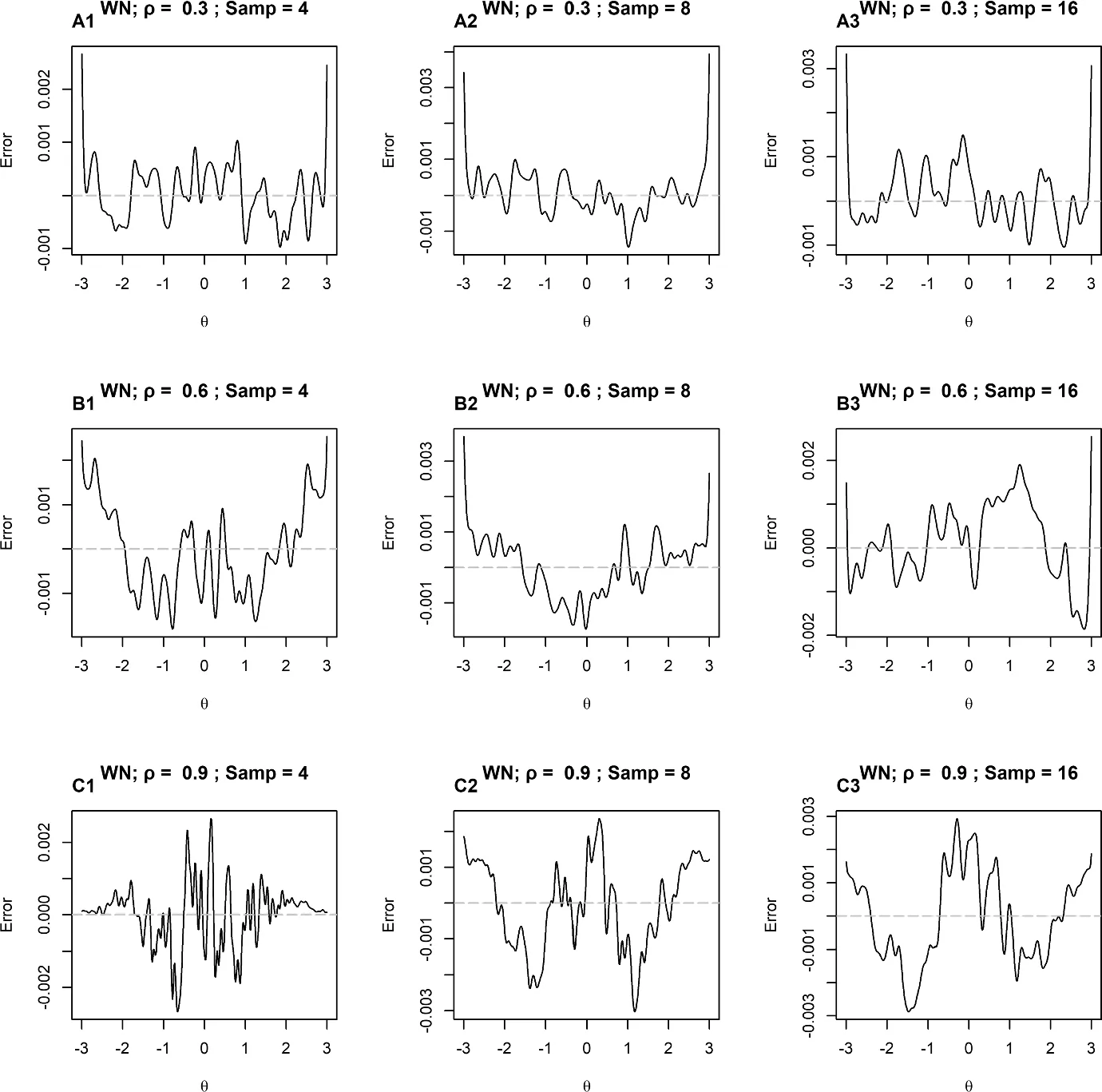
Error plots comparing the predicted distribution curves against the simulated distributions as shown in Fig. 7 in the Main text. Each panel shows the pointwise error between the empirical turning angle density obtained from an initial simulated correlated random walk using a wrapped normal distribution evaluated across $\theta \in [-\pi ,\pi ]$. Results are shown for three concentration parameters ρ=0.3,0.6,0.9, (**A**–**C**) and three sub-sampling rates r=4,8,16 (1–3). Error curves illustrate how approximation accuracy varies with both ρ and sampling rate

**Fig. 19:**
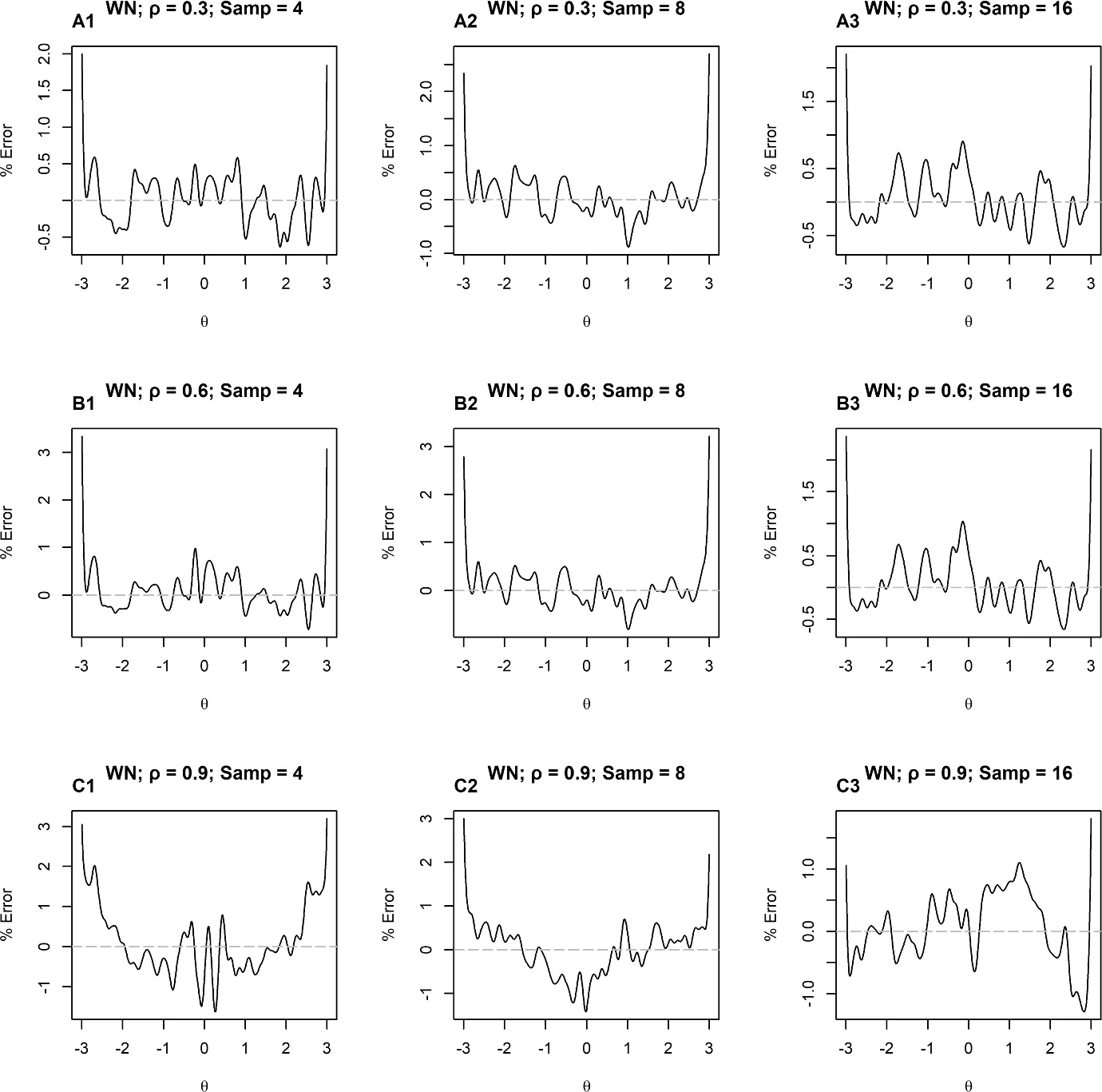
Similar to Fig. 18 except showing the pointwise *percentage* error (*y*-axis)

**Fig. 20:**
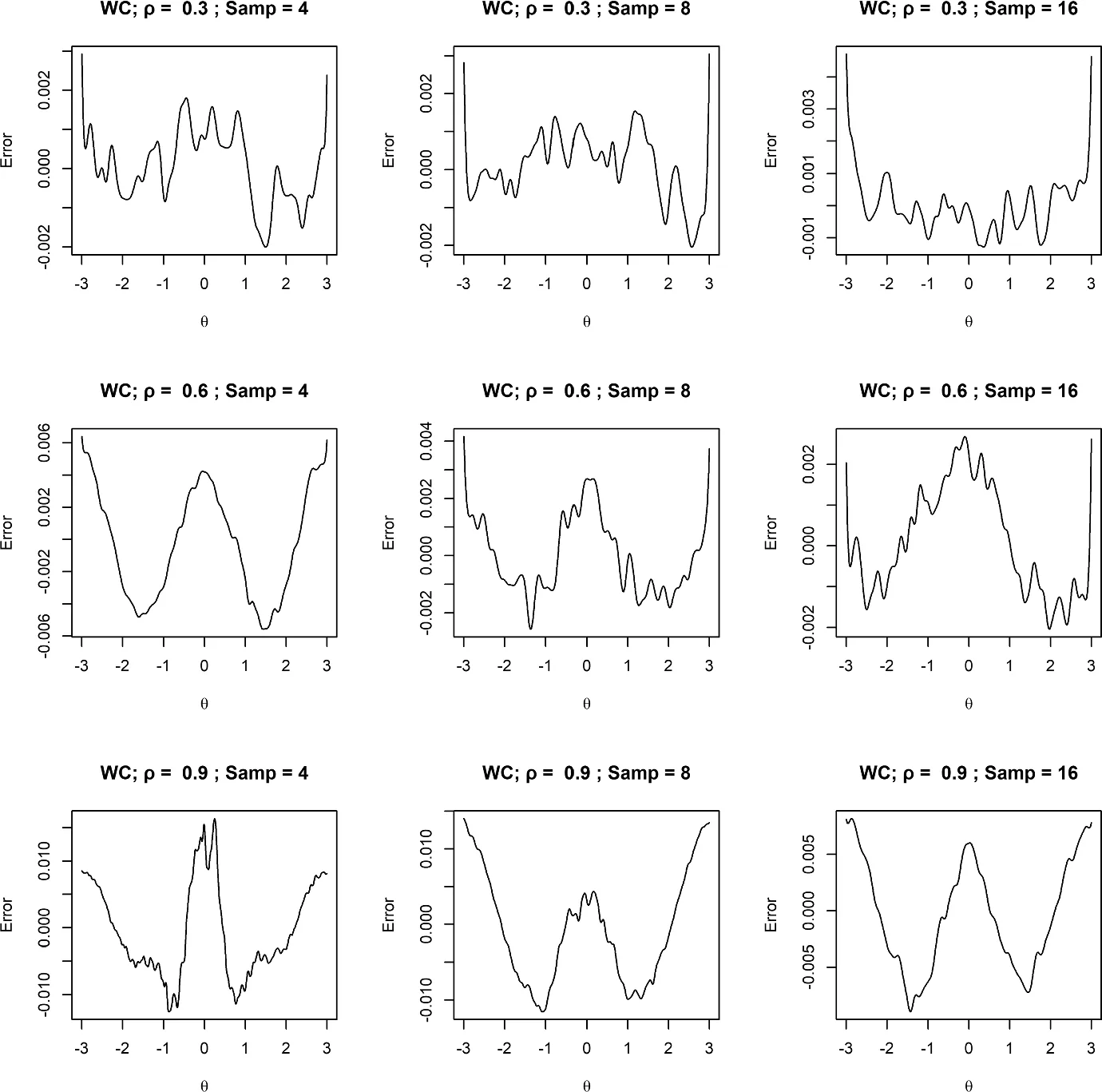
Error plots comparing the predicted distribution curves against the simulated distributions as shown in Fig. 7 in the Main text. Each panel shows the pointwise error between the empirical turning angle density obtained from an initial simulated correlated random walk using a wrapped Cauchy distribution evaluated across $\theta \in [-\pi ,\pi ]$. Results are shown for three concentration parameters ρ=0.3,0.6,0.9, (**A**–**C**) and three sub-sampling rates r=4,8,16 (1–3). Error curves illustrate how approximation accuracy varies with both ρ and sampling rate

**Fig. 21:**
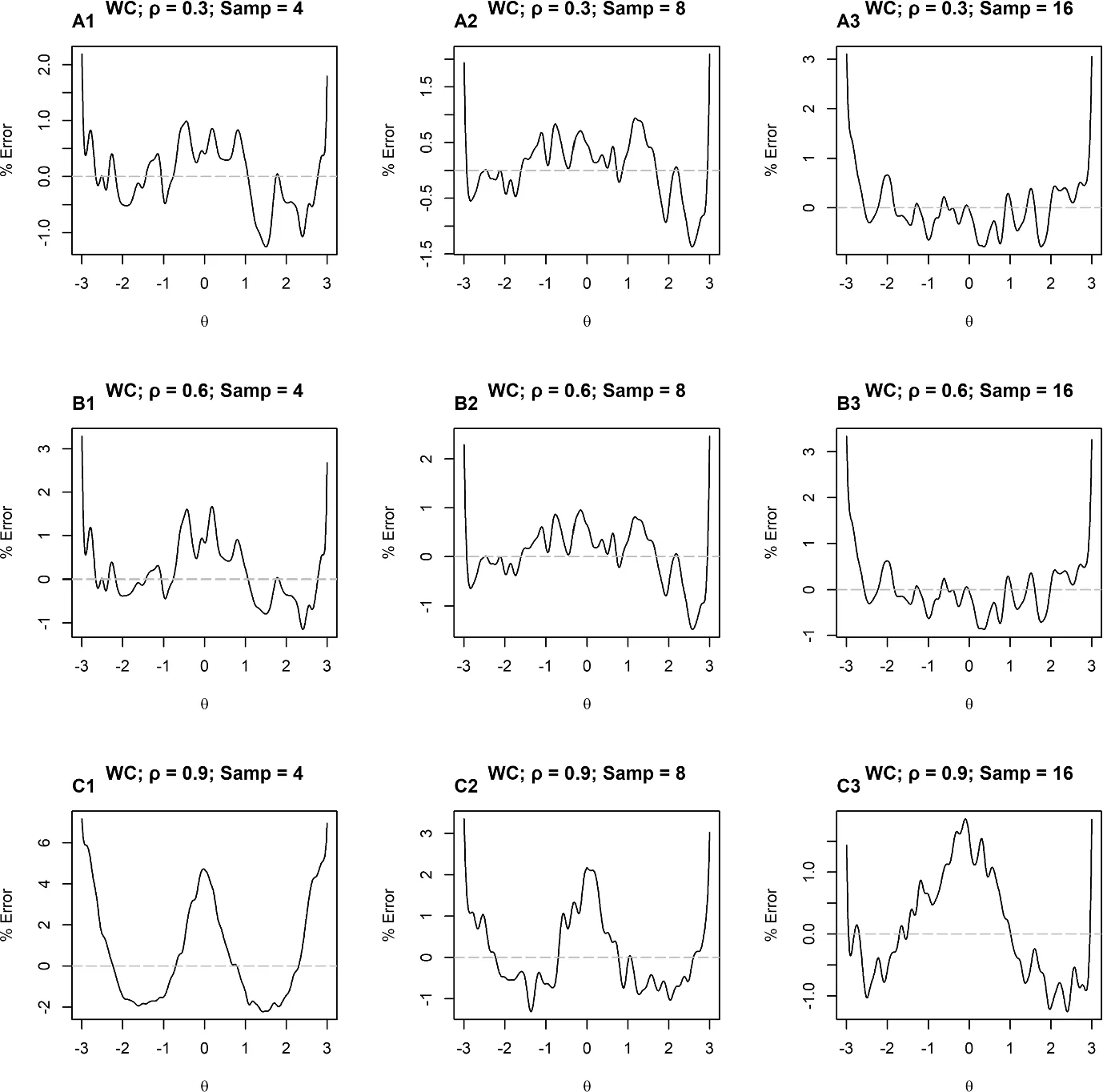
Similar to Fig. 20 except showing the pointwise *percentage* error (*y*-axis)

## Data Availability

The authors declare that the data supporting the findings of this study are available within the paper, and references therein. The R code used to generate the simulations and numerical results is available in the associated GitHub repository. GitHub repository.

## Circular and wrapped distributions

Here we give some brief background notes on wrapped and circular distributions. More information about circular statistics and data can be found in Jammalamadaka and Sengupta (2001); Fisher (1995); Pewsey et al. (2013); Mardia and Jupp (2009) with a helpful short review provided by Lee (2010).

### General information

Circular distributions are probability distributions which have domain $(-\pi ,\pi ]$ and are therefore frequently used in the description of angular data. A distribution, *f*(*X*), can be readily transformed into a circular distribution, $f_\circ (\theta )$ by wrapping it around the unit circle, that is reducing the domain modulo 2π. Giving

A1 $$\begin{aligned} \theta \equiv X\;\mod 2\pi \end{aligned}$$

and allowing the subsequent circular distribution to be considered as

A2 $$\begin{aligned} f_\circ (\theta )=\sum _{m=-\infty }^{\infty }f(\theta +2\pi m),\quad \theta \in (-\pi ,\pi ]. \end{aligned}$$

Distributions which are found by following this wrapping procedure are known as ‘wrapped distributions’ with commonly used examples including the wrapped Cauchy, wrapped normal (Bailey and Codling 2021), but many others are also used in circular data analysis including wrapped exponential, wrapped beta and wrapped generalised Gompertz distribution. Distributions that do not have an equivalent ‘unwrapped’ form are referred to as circular distributions and include the von Mises circular distribution, which is known to closely mimic the wrapped normal distribution (Stephens 1963; Collett and Lewis 1981), and is one of the most commonly used to describe angular data.

### Moments and mean resultant vector (concentration parameter), ρ

An important notion in circular statistics are the *n*th trigonometric moments, $\phi _n$, (which are analogous standard *moments* in probability theory). These are given by

A3 $$\begin{aligned} \phi _n=\textrm{E}[e^{in\theta }]=\int _{-\pi }^{\pi }e^{in\theta }f_\circ (\theta )\,d\theta , \;n\in \mathbb {Z}^+ \end{aligned}$$

alternatively, they can be considered in terms of cosine and sine moments,

$$\begin{aligned} \phi _n&=\textrm{E}[e^{in\theta }]\\\nonumber&=\textrm{E}[\cos {n\theta }+i\sin {n\theta }]\\\nonumber&=\int _{-\pi }^{\pi }\cos {(n\theta )}f_\circ (\theta )\;d\theta +i\int _{-\pi }^{\pi }\sin {(n\theta )}f_\circ (\theta )\;d\theta \\\nonumber&=:\alpha _n+i\beta _n. \end{aligned}$$

The first trigonometric moment, $\phi _1$, which determines the mean of the distribution, can be written as

A4 $$\begin{aligned} \phi _1=\textrm{E}[e^{i\theta }]=\rho e^{i\mu } \end{aligned}$$

where ρ is the length of the mean resultant vector (MRV) and μ the mean direction of the distribution.

In circular statistics, the measure of how ‘peaked’ a distribution is, is given by the length of the mean resultant vector, often also called the *concentration parameter*, which from above we can see is given by

A5 $$\begin{aligned} \rho =\sqrt{\alpha _1^2+\beta _1^2} \end{aligned}$$

where $\alpha _1$ is the mean cosine and $\beta _1$ the mean sine, of the distribution.

### Example wrapped and circular distributions

Here we define a few of the most commonly used wrapped and circular distributions.

Circular uniform distribution

The circular equivalent to the uniform distribution is given by

A6 $$\begin{aligned} f_{\text {U}}(\theta )=\frac{1}{2\pi },\quad \theta \in (-\pi ,\pi ]. \end{aligned}$$

This equates to all angles being equally likely to be chosen and is the turning angle distribution which corresponds to directionless movement as seen in a simple random walk, Brownian motion and Lèvy walks (Codling et al. 2008; Ahmed et al. 2023).

Symmetric wrapped stable distribution

A symmetric wrapped stable (SWS) distribution is a family of wrapped distributions given by the density function:

A7 $$\begin{aligned} f_{\text {sws}}(\theta ;\rho ,\mu ,a)=\frac{1}{2\pi }\left( 1+2\sum _{n=1}^{\infty }\rho ^{n^a}\cos {n(\theta -\mu )}\right) ,\;\;n\in \mathbb {N},\quad \theta \in (-\pi ,\pi ] \end{aligned}$$

where $\rho \in [0,1)$ is the MRV/concentration parameter, $\mu \in [-\pi ,\pi )$ is the mean location parameter around which the distribution is symmetric and a∈(0,2] (Jammalamadaka and Sengupta 2001; Bailey and Codling 2021). SWS distributions are unimodal distributions, which are symmetric around the mean value of μ.

Wrapped Normal distribution

The wrapped normal (WN) distribution is found by taking the standard normal distribution on the reals, and wrapping it around the unit circle as in Eq. A2. This can be represented as

A8 $$\begin{aligned} f_{\text {WN}}(\theta ;\rho ,\mu )=\frac{1}{2\pi }\left( 1+2\sum _{n=1}^{\infty }\rho ^{n^2}\cos {n(\theta -\mu )}\right) ,\;\;n\in \mathbb {N},\quad \theta \in (-\pi ,\pi ] \end{aligned}$$

where $\rho \in [0,1)$ and $\mu \in [-\pi ,\pi )$ are as described for the SWS distribution. Note, this is precisely the from of a SWS with a=2, which demonstrates that the wrapped normal is a SWS distribution.

Wrapped Cauchy distribution

The wrapped Cauchy (WC) distribution is found by taking the standard Cauchy distribution on the reals, and wrapping it around the unit circle as in Eq. A2. Which can be represented as

A9 $$\begin{aligned} f_{\text {WC}}(\theta ;\rho ,\mu )=\frac{1}{2\pi }\left( 1+2\sum _{n=1}^{\infty }\rho ^{n}\cos {n(\theta -\mu )}\right) ,\;\;n\in \mathbb {N},\quad \theta \in (-\pi ,\pi ] \end{aligned}$$

where $\rho \in [0,1)$ and $\mu \in [-\pi ,\pi )$ are as described for the SWS distribution. Note, this is precisely the from of a SWS with a=1, which demonstrates that like the WN, the wrapped Cauchy is also an SWS distribution. However, unlike the WN, the WC can be written in closed form as

A10 $$\begin{aligned} f_{\text {WC}}(\theta ;\rho ,\mu )=\frac{1}{2\pi }\frac{1-\rho ^2}{1+\rho ^2-2\rho \cos {(\theta -\mu )}},\quad \theta \in (-\pi ,\pi ]. \end{aligned}$$

In general, compared with the WN, the WC features a more sharply peaked distribution around the mean value μ, which decays quicker away from the mean but keeping ‘fatter-tails’ at the extreme values (Bailey and Codling 2021).

von Mises distribution

The von Mises (vM) distribution is a circular distribution which does not have an ‘un-wrapped’ equivalent. It is given by the density function,

A11 $$\begin{aligned} f_{\text {vM}}=\frac{1}{2\pi I_0(\kappa )}e^{\kappa \cos (\theta -\mu )},\quad \theta \in (-\pi ,\pi ] \end{aligned}$$

where $\mu \in [-\pi ,\pi )$, κ>0 and $I_0(\kappa )$ is the modified Bessel function of the first kind with order 0. Here, κ acts similarly to ρ in the SWS distributions, however it is unbounded above. The distribution is symmetric around the value of μ and, whilst is not a member of the SWS family of distributions, it closely mimics the properties of the WN (Codling et al. 2010; Collett and Lewis 1981; Stephens 1963). Due to this, and as it can be written in closed form, it is often used in place of the WN.

### Triangle distribution

Here we give details regarding the form of the triangle distribution used in the manuscript; note other forms of a circular triangle distribution exist e.g. Jammalamadaka and Sengupta (2001); Agostinelli and Lund (2022).

The triangle distribution can be considered as a circular distribution in various ways, for example it could be calculated as a wrapped distribution similar to the wrapped Cauchy and wrapped normal where the wrapped triangle distribution is found by taking the triangle distribution on the reals and taking the angles modulo 2π. Here we take the usual triangle distribution on the reals and remove any angles with absolute value greater than π. The resulting density is then re-scaled to give a PDF as shown in Fig. 14 and formulated below.

Let *f*(*x*) be the triangle distribution on the reals that is symmetric around the point $c\in \mathbb {R}$, given by

$$\begin{aligned} f(x;a,c)=\frac{(a-c)-|c-x|}{(a-c)^2} \end{aligned}$$

where $a\in \mathbb {R}_{>0}$. When the distribution is zero centred we have c=0, giving

$$\begin{aligned} f(x;a)=\frac{a-|x|}{a^2} \end{aligned}$$

Essentially, this gives a distribution with domain [-a,a]. If we consider this distribution with domain $[-\pi ,\pi ]$, to give a ‘circular’ triangle distribution, which we denote by $f_\circ$, then in the case where $a\le \pi$ we have

$$\begin{aligned} f_\circ (x;a)={\left\{ \begin{array}{ll}f(x;a), \quad \text {for }x\in [-a,a]\\ 0,\qquad \text {otherwise.} \end{array}\right. } \end{aligned}$$

For any a>π we simply restrict *x* to be in $[-\pi ,\pi ]$ and re-scale. That is, if a>π we have

$$\begin{aligned} f_\circ (x;a)=\frac{f(x;a)}{\left( \int _\pi ^a f(\tilde{x})d\tilde{x}+\int _ {-a}^{-\pi } f(\tilde{x})d\tilde{x}\right) } = \frac{f(x;a)}{F(|x|>a)}=\frac{f(x;a)}{2F(x>a)} \end{aligned}$$

for all $x\in [-\pi ,\pi ]$. This still holds for the case when a<π, hence the circular form of the triangle distribution is therefore given by

$$\begin{aligned} f_\circ (x;a)={\left\{ \begin{array}{ll}{\frac{f(x;a)}{2F(x>a)}}, \quad \text {for }x\in [-a,a]\\ 0,\qquad \text {otherwise.} \end{array}\right. } \end{aligned}$$

where $$f(x;a)=\frac{a-|x|}{a^2}$$.

The mean resultant vector for these distributions for various values of *a* are given in Table 1 following the procedure outlined in Chapter 1 of Jammalamadaka and Sengupta (2001).

**Table 1:** Table showing the value for *a* used in the circular triangle distribution and the corresponding mean resultant vector length ($|\text {MRV}|=\rho$)

*a*	$|\text {MRV}|=\rho$
0.3141593	0.9918025
0.6283185	0.967536
0.9424778	0.9280932
1.2566371	0.8750400
1.5707963	0.8105887
1.8849556	0.7370167
2.1991149	0.6567193
2.5132741	0.5730016
2.8274334	0.4881268
3.1415927	0.4054206
3.4557519	0.3376897
3.7699112	0.2896437
4.0840704	0.2531472
4.3982297	0.2252001
4.7123890	0.2028957
5.0265482	0.1841670
5.3407075	0.1691151
5.6548668	0.1560478
5.9690260	0.1445165
6.2831853	0.1344017

## Example of sampling a CRW at rate r=4

As a worked example of the proof approach of Theorem 2, we consider sampling a CRW at rate $r=2^2=4$ equivalent to k=2. We first follow Eq. 22 to find the coefficients $a^{\{2^2\}}_{t-\tau }$ and then the approximated coefficients $\tilde{a}^{\{2^2\}}_{t-\tau }$ using Eq. 23, recalling that we assume $\sigma _i\sim \textrm{Rad}(p)$ for all *i*.

From Eqs. 18 & 19, with k=2, we have that at point $X_t$ the turning angle of the sub-sampled random walk is

$$\begin{aligned} \theta ^{\{2^2\}}_{X_t}=\sigma _1\theta ^{\{2^1\}}_{X_t}+\sigma _2\alpha ^{\{2^2\}}_{X^-_t}+\sigma _3\alpha ^{\{2^2\}}_{X^+_t} \end{aligned}$$

with

$$\begin{aligned} \alpha ^{\{2^2\}}_{X^-_t}\approx \frac{1}{2}\theta ^{\{2^1\}}_{X_{t-2^1}},\quad \alpha ^{\{2^2\}}_{X^+_t}\approx \frac{1}{2}\theta ^{\{2^1\}}_{X_{t+2^1}}. \end{aligned}$$

Hence

B12 $$\begin{aligned} \theta ^{\{2^2\}}_{X_t}\approx \sigma _1\theta ^{\{2^1\}}_{X_t}+\frac{\sigma _2}{2}\theta ^{\{2^1\}}_{X_{t-2}}+\frac{\sigma _3}{2}\theta ^{\{2^1\}}_{X_{t+2}}. \end{aligned}$$

Now considering $\theta ^{\{2^1\}}_{X_t}$ we have

$$\begin{aligned} \theta ^{\{2^1\}}_{X_{t}}=\sigma _4\theta ^{\{2^0\}}_{X_{t}}+\sigma _5\alpha ^{\{2^1\}}_{X^-_{t}}+\sigma _6\alpha ^{\{2^1\}}_{X^+_{t}} \end{aligned}$$

with

$$\begin{aligned} \alpha ^{\{2^1\}}_{X^-_t}\approx \frac{1}{2}\theta ^{\{2^0\}}_{X_{t-2^0}},\quad \alpha ^{\{2^1\}}_{X^+_t}\approx \frac{1}{2}\theta ^{\{2^0\}}_{X_{t+2^0}}. \end{aligned}$$

Employing the notation convention outlined in Sect. 3.2 that $\theta _i^{\{2^0\}}=\theta ^{\{1\}}_i{\mathop {=}\limits ^{\text {def}}}\theta _i$, gives

B13 $$\begin{aligned} \theta ^{\{2^1\}}_{X_{t}}\approx \sigma _4\theta _{X_{t}}+\frac{\sigma _5}{2}\theta _{X_{t-1}}+\frac{\sigma _6}{2}\theta _{X_{t+1}}. \end{aligned}$$

Similarly, we have

B14 $$\begin{aligned} \theta ^{\{2^1\}}_{X_{t-2}}\approx \sigma _7\theta _{X_{t-2}}+\frac{\sigma _8}{2}\theta _{X_{t-1}}+\frac{\sigma _9}{2}\theta _{X_{t-3}}, \end{aligned}$$

and

B15 $$\begin{aligned} \theta ^{\{2^1\}}_{X_{t+2}}\approx \sigma _{10}\theta _{X_{t+2}}+\frac{\sigma _{11}}{2}\theta _{X_{t+1}}+\frac{\sigma _{12}}{2}\theta _{X_{t+3}}. \end{aligned}$$

Finally, substituting (B13)–(B15) into (B12) gives

$$\begin{aligned}&\theta ^{\{2^2\}}_{X_t}\approx \sigma _1 \left( \sigma _4\theta _{X_{t}}+\frac{\sigma _5}{2}\theta _{X_{t-1}}+\frac{\sigma _6}{2}\theta _{X_{t+1}} \right) + \frac{\sigma _2}{2} \left( \sigma _7\theta _{X_{t-2}}+\frac{\sigma _8}{2}\theta _{X_{t-1}}+\frac{\sigma _9}{2}\theta _{X_{t-3}}\right) \\&\quad + \frac{\sigma _3}{2} \left( \sigma _{10}\theta _{X_{t+2}}+\frac{\sigma _{11}}{2}\theta _{X_{t+1}}+\frac{\sigma _{12}}{2}\theta _{X_{t+3}} \right) \\&\quad =\frac{\sigma _2\sigma _9}{4}\theta _{X_{t-3}} + \frac{\sigma _2\sigma _7}{2}\theta _{X_{t-2}} + \frac{2\sigma _1\sigma _5+\sigma _2\sigma _8}{4}\theta _{X_{t-1}} + \sigma _1\sigma _4 \theta _{X_t} \\&\quad +\frac{2\sigma _1\sigma _6+\sigma _3\sigma _{11}}{4}\theta _{X_{t+1}} + \frac{\sigma _3\sigma _{10}}{2}\theta _{X_{t+2}} + \frac{\sigma _3\sigma _{12}}{4}\theta _{X_{t+3}}. \end{aligned}$$

**Table 2:** Table detailing the step-length distributions used here. Including their respective means and variance. †The colours listed here correspond to the colour of the curves used in Figs. 16 and 17 below

Distribution	Mean	Variance	${\textrm{Line colour}}^\dagger$
Fixed (ℓ=1)	1	0	Red
Uniform (min=0, max=1000)	500.5	83,167	Black
Exp(λ = 0.5)	2	4	Cyan
Exp(λ = 1)	1	1	Green
Exp(λ = 2)	0.5	0.25	Magenta
Log-normal($\mu =0, \sigma =1$)	1.649	4.671	Blue
Log-normal($\mu =0, \sigma =0.5$)	1.133	0.365	Gold
Log-normal($\mu =0.5, \sigma =1$)	2.718	12.696	Silver
Log-normal($\mu =1, \sigma =1$)	4.482	34.513	Dark grey
Log-normal($\mu =1, \sigma =0.5$)	3.080	2.695	Rose
Log-normal($\mu =2, \sigma =0.5$)	8.373	19.912	Light-green

Thus $\theta ^{\{2^2\}}_{X_t}$ is written in terms of the turning angles from the original CRW $\theta _{X_{t-\tau }}$ with $\tau =\{-3,-2,\ldots ,3\}$, and the random variables $\sigma _i\sim \textrm{Rad}(p)$, i=1,⋯,12, (see Fig. 15 for graphical representation).

Following the rules in Eq. 23 to give the coefficients $\tilde{a}^{\{2^2\}}_{t+\tau }$ we get the below expression

B16 $$\begin{aligned} \theta ^{\{2^2\}}_{X_t}=(2p-1)^2\left( \frac{1}{4}\theta _{X_{t-3}}+\frac{2}{4}\theta _{X_{t-2}}+\frac{3}{4}\theta _{X_{t-1}}+\theta _{X_t}+\frac{3}{4}\theta _{X_{t+1}}+\frac{2}{4}\theta _{X_{t+2}}+\frac{1}{4}\theta _{X_{t+3}}\right) \end{aligned}$$

This allows the distribution of $\theta ^{\{2^2\}}_{X_t}$, denoted by $f^{\{4\}}$, to be calculated by convolution, giving;

$$\begin{aligned} f^{\{4\}}(\theta )&= \frac{4}{(2p-1)^2} f_\circ \!\left( \frac{4\theta }{(2p-1)^2}\right) *\frac{4}{2(2p-1)^2} f_\circ \!\left( \frac{4\theta }{2(2p-1)^2}\right) \\&\quad *\frac{4}{3(2p-1)^2} f_\circ \!\left( \frac{4\theta }{3(2p-1)^2}\right) *\frac{1}{(2p-1)^2} f_\circ \!\left( \frac{\theta }{(2p-1)^2}\right) \\&\quad *\frac{4}{3(2p-1)^2} f_\circ \!\left( \frac{4\theta }{3(2p-1)^2}\right) *\frac{4}{2(2p-1)^2} f_\circ \!\left( \frac{4\theta }{2(2p-1)^2}\right) \\&\quad *\frac{4}{(2p-1)^2} f_\circ \!\left( \frac{4\theta }{(2p-1)^2}\right) \\&= \frac{4^6}{(3\cdot 2\cdot 1)^2} \frac{1}{\bigl ((2p-1)^2\bigr )^7} f_\circ \!\left( \frac{\theta }{(2p-1)^2}\right) *f_\circ ^{*2}\!\left( \frac{4\theta }{(2p-1)^2}\right) \\&\quad *f_\circ ^{*2}\!\left( \frac{4\theta }{2(2p-1)^2}\right) *f_\circ ^{*2}\!\left( \frac{4\theta }{3(2p-1)^2}\right) \\&= \frac{\left( p-\frac{1}{2}\right) ^{-14}}{(3\cdot 2\cdot 1)^2} f_\circ \!\left( \frac{\theta }{(2p-1)^2}\right) *f_\circ ^{*2}\!\left( \frac{4\theta }{(2p-1)^2}\right) \\&\quad *f_\circ ^{*2}\!\left( \frac{4\theta }{2(2p-1)^2}\right) *f_\circ ^{*2}\!\left( \frac{4\theta }{3(2p-1)^2}\right) . \end{aligned}$$

which we note is equivalent to the result given by Theorem 2 with k=2.

Finally, transforming this into a distribution on the unit circle gives the final distribution,

B17 $$\begin{aligned} f^{\{4\}}_\circ (\theta )=\sum _{k=-(2^4-1)}^{2^4-1} f^{\{4\}}\left( \theta \pm 2k\pi \right) . \end{aligned}$$

## Step length distribution doesn’t affect turning angle distribution

Here we show that the choice of distribution for the step lengths (Λ) will not affect the resulting distributions of the turning angles from sub-sampled random walks, under the assumption that the distribution describing the step lengths is unimodal or uniform.

Figures 16 and 17 below demonstrate that various differing initial step-length distributions, including those with large variances, give very similar results in the turning angle distributions of the sub-sampled random walk. This indicates that the initial step-length does not have a significant effect on the expected turning angle distribution. Note, all this work concerns distributions with finite variances and so Lèvy walk and power-law behaviour is not considered.

## Computing the coefficients $\tilde{a}^{\{2^{k}\}}_{t+\tau }$

Recall for $\tau =-(2^k-1),\dots ,2^k-1$ the coefficients $\tilde{a}^{\{2^{k}\}}_{t+\tau }$ are defined within the proof of Theorem 2, where $\tilde{a}^{\{2^{k}\}}_{t+\tau }=\tilde{a}^{\{2^{k}\}}_{t-\tau }$ and for $\tau =0,\dots ,2^k-1$:

D18 $$\begin{aligned} \tilde{a}^{j}_{t\pm \tau }= \textrm{E}\left[ a^{j}_{t+\tau }\right] = {\left\{ \begin{array}{ll} (2p-1)\tilde{a}^{j-1}_{t+\frac{\tau }{2}},\quad \tau \text { even},\\ \frac{1}{2}(2p-1)\tilde{a}^{j-1}_{t+(\frac{\tau -1}{2})},\quad \tau =2^{j}-1\\ \frac{1}{2}(2p-1)\left[ \tilde{a}^{j-1}_{t+\frac{\tau -1}{2}}+\tilde{a}^{j-1}_{t+\frac{\tau +1}{2}}\right] ,\; \text {else} \end{array}\right. } \end{aligned}$$

(Eq. 23 in the Main Text). Here we prove $\tilde{a}^{\{2^{k}\}}_{t+\tau }$ can be written equivalently as:

D19 $$\begin{aligned} \tilde{a}^{\{2^k\}}_{t+\tau }=\frac{2^k-|\tau |}{2^k}(2p-1)^k, \end{aligned}$$

where k≥0, and $$\tau =-(2^k-1),\ldots ,2^k-1$$.

This can be shown through induction. The case k=0 follows trivially from Eq. D18, since $\tilde{a}^{\{1\}}_t=\tilde{a}^0_t=1$ and substituting k=0 in Eq. D19 gives $\tilde{a}^0_{t+\tau }=1-\tau$, where τ can only take value $(2^0-1)=0$.

We proceed with induction on *k*. Assume Eq. D19 holds for *k*, now consider the expression for k+1. Without loss of generality (since $\tilde{a}^{\{2^{k}\}}_{t+\tau }=\tilde{a}^{\{2^{k}\}}_{t-\tau }$) we assume $\tau \ge 0$. Working through each of the three cases separately, firstly take τ to be even, case (i), then by Eq. D18

$$\begin{aligned} \tilde{a}^{\{2^{k+1}\}}_{t+\tau }=\tilde{a}^{k+1}_{t+\tau }&=(2p-1)\tilde{a}^{k}_{t+\frac{\tau }{2}} \\ \nonumber&=(2p-1)\frac{2^k-|\tau /2|}{2^k}(2p-1)^k \\ \nonumber&= \frac{2^{k+1}-|\tau |}{2^{k+1}}(2p-1)^{k+1}, \end{aligned}$$

as required. Next, case (ii), assume $\tau =2^{k+1}-1$ and note that for this,

$$\begin{aligned} \frac{\tau -1}{2}&=\frac{2^{k+1}-2}{2} \\ \nonumber&=2^k-1. \end{aligned}$$

Therefore for $\tau =2^{k+1}-1$ and by Eq. D18, we have

$$\begin{aligned} \nonumber \tilde{a}^{\{2^{k+1}\}}_{t+\tau }=\tilde{a}^{k+1}_{t+\tau }&= \frac{1}{2}(2p-1)\tilde{a}^{k}_{t+(\frac{\tau -1}{2})} \\ \nonumber&=\frac{1}{2}(2p-1) \frac{2^k-|(\tau -1)/2|}{2^k}(2p-1)^k \\ \nonumber&= \frac{2^k-(2^k-1)}{2^{k+1}}(2p-1)^{k+1} \\ \nonumber&= \frac{2^{k+1}-(2^{k+1}-1)}{2^{k+1}}(2p-1)^{k+1} \\ \nonumber&= \frac{2^{k+1}-|\tau |}{2^{k+1}}(2p-1)^{k+1}, \end{aligned}$$

as required. Finally, we consider the case of odd $\tau <2^{k+1}-1$. Applying Eq. D18,

$$\begin{aligned} \tilde{a}^{\{2^{k+1}\}}_{t+\tau }&= \frac{1}{2}(2p-1)\left[ \tilde{a}^{k}_{t+\frac{\tau -1}{2}}+\tilde{a}^{k}_{t+\frac{\tau +1}{2}}\right] \\&=\frac{1}{2}(2p-1)\left[ \frac{2^k- |(\tau -1)/2|}{2^k}(2p-1)^k + \frac{2^k-|(\tau +1)/2|}{2^k}(2p-1)^k \right] \\&=\frac{1}{2^{k+1}}\left[ 2^k-\frac{\tau -1}{2} +2^k-\frac{\tau +1}{2} \right] (2p-1)^{k+1} \\&=\frac{2^{k+1}-|\tau |}{2^{k+1}}(2p-1)^{k+1}. \end{aligned}$$

Thus in all three cases, given the inductive hypothesis, Eq. D19 holds for k+1, therefore the claim holds for all k≥0.

## Error plots for Fig. 7

Here we give error plots for the difference between the predicted results given by Theorem 2 and those found through extensive simulations, as shown in Fig. 7.

The pointwise error (Figs. 18 and 20) measures, at each angle θ, the direct difference between the empirical turning angle density obtained from the simulated correlated random walk and the theoretical density predicted by Theorem 2.

The pointwise percentage error (Figs. 19 and 21) calculates the percentage deviation between the observed and theoretical results across θ.
